# Integrative discovery and functional dependency mapping uncover essential chimeric RNAs in the active transcriptome

**DOI:** 10.1126/sciadv.aei0901

**Published:** 2026-10-30

**Authors:** Samir Lalani, Samuel Haddox, Kendall Bromley, Shafaque Zahra, Marina Wang, Mason Ingram, Sehajroop Gadh, Shriya Gunalan, Sulun Ke, Qiong Wang, Fujun Qin, Eric Hendrickson, Anja Bielinsky, Hui Li

**Affiliations:** ^1^ Department of Biochemistry and Molecular Genetics, University of Virginia, Charlottesville, VA 22908, USA.; ^2^ Department of Pathology, University of Virginia, Charlottesville, VA 22908, USA.; ^3^ Department of Urology, Nanfang Hospital, Southern Medical University, Guangzhou, Guangdong 510515, China.; ^4^ State Key Laboratory of Metabolic Dysregulation & Prevention and Treatment of Esophageal Cancer, Tianjian Laboratory of Advanced Biomedical Sciences, School of Convergence Medicine, Zhengzhou University, Zhengzhou 450052, China.; ^5^ Department of Medicine, University of Virginia, Charlottesville, VA 22908, USA.; ^6^ Comprehensive Cancer Center, University of Virginia, Charlottesville, VA 22908, USA.; ^7^ Center for RNA Science and Medicine, University of Virginia, Charlottesville, VA 22908, USA.

## Abstract

Chimeric RNAs are a class of molecules defined as RNA transcripts containing genetic information for multiple genes. While their expression patterns across tissue types and disease states have been well documented, the biological function of these transcripts remains poorly understood. In this study, we developed an integrative prediction pipeline to establish an atlas of 569,640 chimeric RNAs across 1019 cancer cell lines. Leveraging this resource, we performed a systematic functional dependency screen by integrating our predictions with perturbation datasets from The Cancer Dependency Map project. This approach moves beyond prediction to identifying high-confidence functional chimeric RNAs essential for cellular processes, including a subset of recurrent chimeric transcripts generated by cis*-*splicing of adjacent genes. We demonstrate that depletion of selected candidate chimeras disrupts cell cycle progression and cellular fitness and that these transcripts are frequently dysregulated in primary breast tumors. Together, these results demonstrate that chimeric RNAs constitute a previously underappreciated layer of gene expression, expanding the functional genome beyond conventional genetics.

## INTRODUCTION

Phenotypic diversity across organisms counterintuitively does not correlate with the number of protein-coding genes, a phenomenon frequently termed the *G*-value paradox (*1*). This discrepancy is partially resolved by transcriptomic complexity, which expands the functional capabilities of the genome through mechanisms such as alternative splicing and posttranscriptional modifications (*2*, *3*). These processes enable individual genetic loci to encode multiple distinct functional units, thereby expanding functional diversity contributing to biologic complexity. However, despite the recognition of these mechanisms, the full extent of transcriptomic diversity remains unknown.

An underexplored contributor to transcriptomic diversity is chimeric RNA, defined as transcripts composed of exons from two or more distinct genes (*4*). Initial studies into chimeric RNAs have focused on their relationship with chromosomal rearrangements in cancer, while chimeric transcripts found in normal physiology were historically disregarded as technical artifacts or nonfunctional transcriptional noise (*5*). However, chimeric RNAs are now recognized as bona fide RNA transcripts and widespread in normal physiology, found across all tissues and developmental states (*6*–*8*). These chimeric RNAs arise through multiple mechanisms, including DNA-level structural variations and RNA-level processes, such as cis–splicing of adjacent genes (cis*-*SAGe) and RNA trans-splicing (*9*–*13*). By creating unique exon combinations, chimeric RNAs can generate unique coding sequences or noncoding transcripts, effectively expanding the functional genome beyond canonical gene boundaries.

Dysregulation of chimeric RNAs play a crucial role in disease biology, particularly cancer. DNA rearrangements leading to the expression of chimeric RNAs encoding oncogenic fusion proteins have long been established as hallmarks of cancer. The discovery of DNA-level oncogenic gene fusions such as *BCR-ABL1* (chronic myeloid leukemia) and *EML4-ALK* (lung adenocarcinoma) has led to successful targeted therapies, revolutionizing the way these cancers are treated (*14*–*16*). More recently, RNA-level fusions, particularly those derived from cis*-*splicing of adjacent genes, have also been shown to play a role in specific cancers. These include *SLC45A3-ELK4* and *D2HGDH-GAL3ST2* (prostate cancer) (*10*, *17*, *18*), *C19orf47-AKT2* (glioma) (*19*), *LHX6-NDUFA8* (cervical cancer) (*20*), and *BCL2L2-PABPN1* (bladder cancer) (*21*).

Despite their prevalence, the biological significance of most chimeric RNAs remains unclear, with the majority lacking functional characterization. Distinguishing biologically relevant chimeric RNAs from transcriptional noise is a major challenge, particularly for RNA-derived events that may exhibit low expression and may not produce obvious protein products. This knowledge gap presents an opportunity to uncover previously unexplored dimensions of gene expression and regulation by investigating chimeric RNAs and their contributions to the functional genome.

Functional dependency mapping offers a powerful strategy to address this challenge. Large-scale perturbation screens using RNAi or CRISPR-Cas9, such as those from the Cancer Dependency Map project (DepMap), systematically measure the impact of gene perturbation on cell viability across hundreds of cancer cell lines (*22*–*25*). Integrating fusion predictions with these functional datasets enables the identification of fusions that contribute to cell fitness, as demonstrated by previous studies leveraging CRISPR-Cas9 data to evaluate oncogenic gene fusions (*26*, *27*). However, while CRISPR-Cas9–based approaches are effective for investigating DNA-level gene fusions, the whole-gene knockout simultaneously affects both the chimeric RNA and parental transcripts in the same loci, making it difficult to isolate chimera-specific essentiality from that of the parental genes. Accordingly, RNAi enables perturbation targeted to specific loci along the transcript without altering the underlying DNA, providing higher-resolution screening and providing us a practical framework for chimeric RNA dependency mapping.

In this study, we generated chimeric RNA predictions for 1019 cancer cell lines and integrated them with transcriptome-wide RNAi dependency screening data to systematically characterize the landscape and functional significance of chimeric RNAs. Our study results not only recapitulate known oncogenic fusions but also reveal a previously uncharacterized class of cis*-*SAGe chimeric transcripts that exhibit diverse regulatory roles that are essential to cellular viability. Furthermore, we demonstrate that dysregulation of these transcripts is enhanced in breast cancer primary tumor samples, underscoring their clinical significance. By integrating discovery and functional profiling, we provide a resource and analytical framework for elucidating the biological impact of chimeric RNAs.

## RESULTS

### Generation of a chimeric RNA atlas across cancer cell lines

To construct a comprehensive database of chimeric RNAs across diverse cellular contexts, we analyzed RNA sequencing (RNA-seq) data from 1019 cancer cell lines representing 26 tissue lineages from the Cancer Cell Line Encyclopedia (CCLE) (Fig. 1A). (*28*). To maximize discovery and mitigate software-specific biases, we used three complementary fusion detection tools, STAR-Fusion, Arriba, and FuSeq, selected based on prior benchmarking for accuracy and precision (*29*–*33*). Given that many programs remove RNA-level chimeras occurring between adjacent genes or at low expression levels by default, we modified default parameters to remove gene distance-based filters and expression cutoffs. In addition, blacklists excluding transcripts reported in normal tissues were also disabled to ensure capture of all physiologically relevant fusion events.

**Fig. 1.**
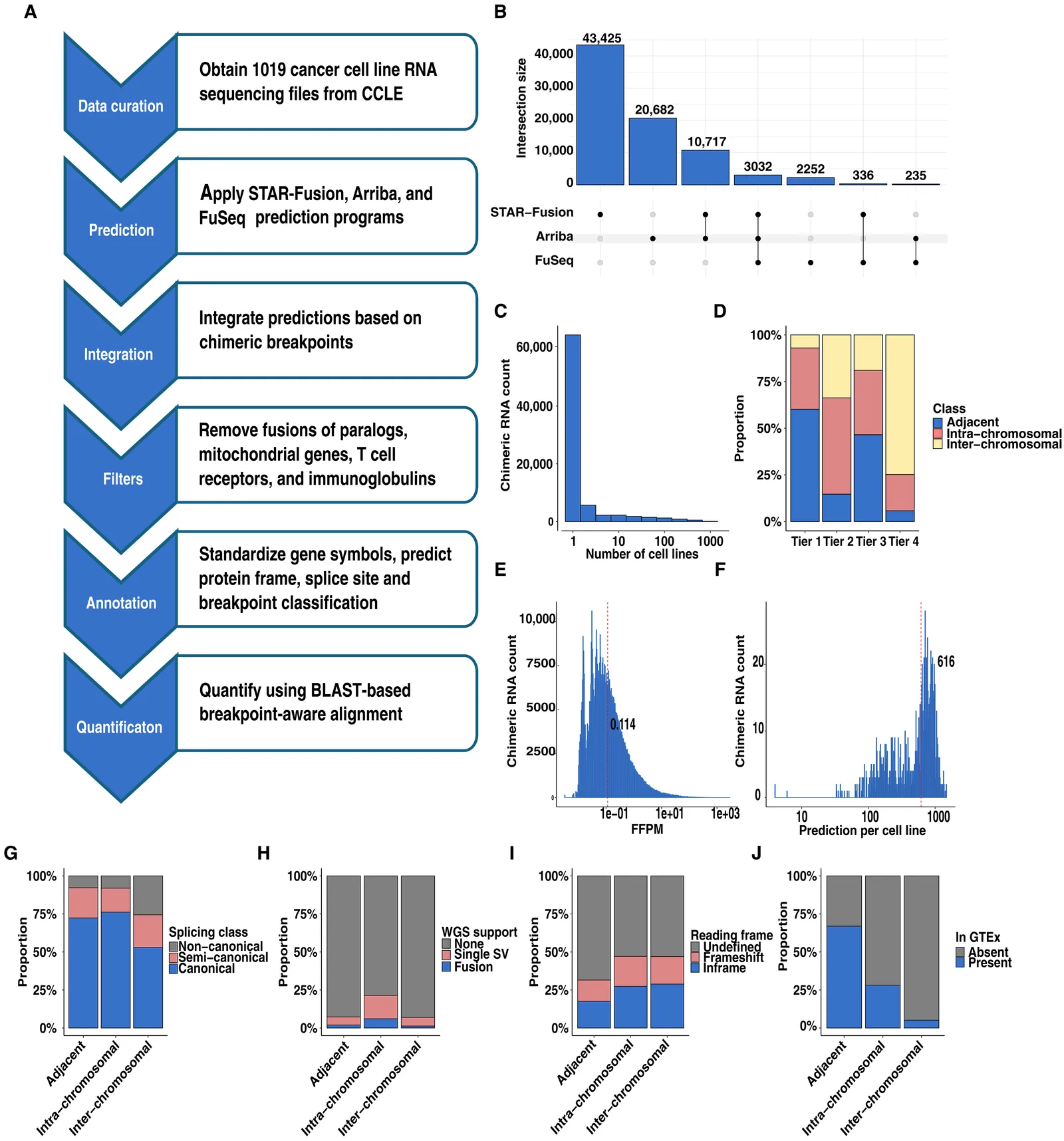
The landscape of chimeric RNAs across cancer cell lines. (**A**) Workflow summarizing the chimeric RNA prediction pipeline applied to 1019 RNA-seq datasets from the Cancer Cell Line Encyclopedia (CCLE). (**B**) Intersection plot of the overlap of chimeric RNA predictions among the three algorithms. Labels on bars show the total number of chimeric RNA predictions in each category across all cell lines. (**C**) Histogram depicting the recurrence of chimeric RNAs, defined as number of cell lines each chimeric RNA appears in across CCLE. (**D**) Comparison of breakpoint classification (adjacent, intrachromosomal, and interchromosomal) across confidence tiers. (**E**) Distribution of chimeric RNA expression levels in FFPM across all predictions. The red line represents median FFPM across all transcripts. (**F**) Distribution of the number of predicted chimeric RNAs per cell line. The red line represents the median number of predicted chimeric RNAs per cell line. (**G**) Splicing classification comparison of chimeric RNAs grouped by breakpoint class. (**H**) DNA-level support for chimeric RNAs based on structural variant (SV) calls from whole-genome sequencing grouped by breakpoint class. (**I**) Proportion of chimeric RNAs predicted to encode in-frame or frameshifted proteins grouped by breakpoint class. (**J**) Proportion of chimeric RNAs previously detected in normal tissues from GTEx grouped by breakpoint class.

Following harmonization of predictions across callers and generation of junction sequences, we applied several filters to enhance the specificity of our predictions. We removed predictions involving mitochondrial genes, as their polycistronic genome architecture and distinct transcriptional mechanisms can confound fusion prediction programs (*34*), as well as immunoglobulins and T cell receptors as they undergo frequent rearrangement and are not the focus of this study. To reduce mapping artifacts, we implemented a paralog filter by constructing a reference of all combinations of paralog genes as annotated in the ENSEMBL database and excluding predictions that matched a potential fusion of high-homology paralogs. In addition, we required all retained chimeric RNAs to exhibit breakpoints located on exons, enriching for processed chimeric transcripts and reducing the likelihood of artifacts arising from unspliced read-through transcription or reverse transcription–mediated template switching.

Quantification of chimeric RNA and parental transcripts was performed with the FusionBlaster program (*35*) by aligning raw sequencing reads against a sample-specific reference library of predicted full-length fusion isoforms and wild-type (WT) parental sequences, reporting raw read counts and fusion fragments per million (FFPM). Following prediction, each chimera was annotated for breakpoint locations, splicing patterns, protein-coding potential, and parental gene biotype. Splicing classification was performed using a bespoke script mapping breakpoints to known exon boundaries, while protein reading frame was assessed using a modified script based on the FusionCatcher program (*36*)

In total, we identified 569,640 chimeric RNA predictions across 1019 cell lines, representing 80,679 unique gene pairs (table S1). Consistent with prior studies, overlap among callers was limited, with only 3032 unique transcripts detected by all three tools (Fig. 1B) (*26*, *37*, *38*). However, as prior studies show that single-caller predictions validate at appreciable rates and we observe these calls constitute a distinct proportion of total predictions, we retained all predictions for downstream analysis (*37*). However, to identify chimeric RNAs with stronger bioinformatic support, we classified them into confidence tiers, with tier 1 (*n* = 5320) showing detection with multiple tools across multiple cell lines, tier 2 (*n* = 8162) detected with multiple tools in a single cell line, tier 3 (*n* = 15,632) being detected across multiple cell lines, but with a single tool, and tier 4 (*n* = 58,778) detected only with a single tool in a single cell line. Most chimeric RNAs were only found in a single cell line (80.1%, *n* = 64,648), but a notable subset was highly recurrent, with the 10 most frequent chimeras present in ≥900 cell lines (Fig. 1C). We categorized chimeric RNAs according to their parental gene breakpoint locations: interchromosomal (different chromosomes), intrachromosomal (same chromosome, different strand, or >70 kb apart), or adjacent (same chromosome, same strand, <70 kb apart). Previous studies often label the adjacent class as “read-through” or “*cis-*SAGe”; however, we adopted the term “adjacent” to remain mechanism-agnostic, as RNA-seq alone cannot distinguish between transcriptional read-through and DNA-level events like microdeletions that may trigger the fusion formation. Our analysis revealed that interchromosomal fusions were the most abundant, followed by intrachromosomal and then adjacent fusions. However, in confidence tiers 1 and 3, which emphasize recurrence, we observe higher levels of adjacent class transcripts (Fig. 1D), showing that adjacent chimeras are more likely to be expressed across different cell lines while inter- and intrachromosomal transcripts are more likely to be cell line specific. Across all samples, the median number of chimeric RNAs per cell line was 616, while the median expression of 0.114 FFPM (Fig. 1, E and F).

To examine how chimeric RNA abundance varied across tissue lineages, we plotted the number of unique predicted chimeric RNA across different tissues. Median counts per cell line ranged from 150 (small intestine) to 763.5 (prostate), with an overall median of 616 chimeric RNAs per cell line (fig. S1A). While a one-way analysis of variance (ANOVA) indicated significant variation (*P* = 0.022), the effect size was small (*F* = 1.68), and no pairwise differences were significant by Tukey’s honest significant difference test. This suggests that tissue of origin has a limited effect on chimeric RNA burden in cancer cell lines, contrasting with the tissue-specific chimeric RNA signatures previously observed in normal GTEx tissues (*6*). This discrepancy likely reflects the widespread splicing dysregulation and karyotypic instability characteristic of immortalized cancer models overshadowing tissue-specific signatures.

One-fifth (20.3%) of predicted chimeric RNAs were found to have multiple breakpoints within a cell line, suggesting the existence of multiple isoforms (fig. S1B). The majority of these only had one additional detected isoform, with lower percentages for each additional isoform. We also observed a drastic drop in expression from the primary isoform to secondary, with the secondary isoform only having around 50% the expression (FFPM) level as the primary. Each successive isoform showed a further decrease (fig. S1C). On the basis of this observation, we surmise that most chimeric RNAs have a primary isoform. Given the fact that our functional screen methodology cannot distinguish between isoforms within a cell line, we focused on the primary isoform, defined by highest FFPM, in our downstream analysis.

We next explored the relationship between the expression of chimeric RNAs and their corresponding parental genes to determine whether chimeras are simply a by-product of high parental gene transcription. Linear regression analysis comparing transcript-specific read counts revealed a low *R*^2^ value in the adjacent and intrachromosomal groups, indicating that parental gene expression is not the primary driver of chimeric RNA abundance; however, this may vary on a per-chimera basis. For interchromosomal fusions, *R*^2^ was slightly higher (5′ *R*^2^ = 0.26, 3′ *R*^2^ = 0.22), indicating a potentially stronger relationship (fig. S1, D and E).

Analysis of splicing and structural features provided further insights into the mechanism of chimeric RNA formation. The majority of chimeric RNAs (61.8%) used canonical splicing (both breakpoints at known exon boundaries), while 19.7% were semicanonical (one breakpoint on known exon boundaries) and 18.4% were noncanonical (neither breakpoint on known exon boundaries). Interchromosomal fusions exhibited the lowest rate of canonical splicing (53.0%) compared to intrachromosomal (76.2%) and adjacent events (72.3%) (Fig. 1G). When looking across confidence classes, tiers 1 and 2 exhibited much higher rates of canonical splicing than tier 3 and tier 4 (fig. S2A).

Structural variant (SV) analysis from whole genome sequencing (WGS) reinforced these class distinctions. WGS support for SVs in at least one parental gene was found in 14.1% of predictions, but exact breakpoint matches occurred in only 3.0%. Support was highest for intrachromosomal fusions (23.4%), intermediate for adjacent-gene chimeras (16.7%), and lowest for interchromosomal events (7.2%) (Fig. 1H). Tier 1 and tier 2 chimeric RNA predictions showed a much higher likelihood of having an underlying SV, particularly tier 2, suggesting that in the case of high-confidence DNA rearrangement–derived fusions, many are rare and only in single cell lines.

To predict whether chimeric RNAs are likely to encode proteins, we compared the positions of chimeric breakpoints to the annotated canonical reading frames of the parental genes. A chimeric RNA was classified as in-frame if both breakpoints fell within annotated coding regions and the 3′ gene maintained its original reading frame, or frameshift if the 3′ breakpoint was in a coding region but disrupted the reading frame. Chimeras where neither breakpoint overlapped a canonical coding region were classified as undefined. Overall, 44.6% of chimeric RNAs were predicted to code defined protein products with 26.8% in-frame and 17.9% frameshift. Adjacent-gene chimeras were notably less likely to contain defined reading frames, with only 31.5% falling into either protein-coding category (Fig. 1I). This was consistent across confidence tiers, with adjacent chimeras consistently being less likely to produce a defined protein product (fig. S2C).

Furthermore, overlap with publicly available chimeric RNA predictions for normal tissue from The Genome Tissue Expression Projects (GTEx) (*6*, *39*) revealed that 20.4% of total predictions were present in normal tissues, though this was highly variable by class: 67.0% of adjacent chimeras were detected in normal samples, compared to only 28.1% of intrachromosomal and 5.1% of interchromosomal events (Fig. 1J). Of note, however, these studies used different prediction pipelines than the ones used in this study, so naturally the two will not have perfect overlap. Among confidence tiers, we see that tiers 1 and 3 have higher overall levels of overlap with GTEx, suggesting that highly recurrent chimeric RNAs are more likely to be found in normal, healthy tissue (fig. S2D).

Analysis of the parental gene biotype of chimeric RNAs revealed a strong bias toward protein-coding genes, comprising 76.4% of 5′ partners and 74.0% of 3′ partners (fig. S2A). While this was noticeably more when compared to the overall transcriptome composition in the CCLE, where only 39.0% of expressed genes are protein-coding, normalizing for expression actually revealed that chimeric RNAs composed of protein-coding genes make up less of the proportion of protein-coding genes than the weighted transcriptome. Overall, fusions between noncoding genes (including pseudogenes, lncRNAs, and antisense transcripts) without a protein-coding gene involved were rare, only making up 2.5% percentage of chimeric RNAs (fig. S2, E and F).

### Functional screen of chimeric RNAs

To investigate the biological significance of the chimeric RNAs, we took advantage of RNAi-based dependency datasets from the Dependency Map project (DepMap), which consists of two pan-cancer RNAi screening projects: Project Drive from Novartis and Project Achilles from the Broad Institute (*22*, *23*). Each gene in these projects is targeted by multiple shRNAs distributed across the transcript, so individual shRNAs frequently produce different dependency scores depending on which transcript segments they target. We reasoned that this probe-level heterogeneity could be exploited to detect fusion-specific essentiality. More specifically, if dependency scores differ for shRNAs positioned on opposite sides of a chimeric breakpoint, this may indicate that the chimeric transcript, rather than the parental gene alone, contributes to cell viability and proliferation.

On the basis of this logic, we developed a systematic functional dependency screen integrating the RNAi-based dependency data with our chimeric RNA predictions. Briefly, shRNA sequences targeting genes found in chimeric RNAs were aligned to the human genome using Bowtie2 (table S2). Each shRNA was then classified based on its position relative to the chimeric breakpoint: chimera-targeting probes (mapping upstream of the chimeric breakpoint for the 5′ gene or downstream of the breakpoint for the 3′ gene) or parent-specific probes. Probes aligning to multiple loci were excluded to ensure specificity. We computed a dependency score per gene to reflect the difference in scaled log_2_ fold change (log_2_FC) between chimera-targeting and parent-specific probes. To estimate statistical significance, we generated 10,000 random permutations of the fold-change values, fit a normal distribution to the resulting null scores, and calculated *P* values from the fitted null distribution (Fig. 2A). Multiple hypothesis testing was controlled using Storey’s *q*-value method within each confidence tier, to account for differences in error structure and artifact levels across tiers and avoiding dilution of high-confidence functional signals by lower-confidence predictions, and the resulting false discovery rates (FDRs) were reported.

**Fig. 2.**
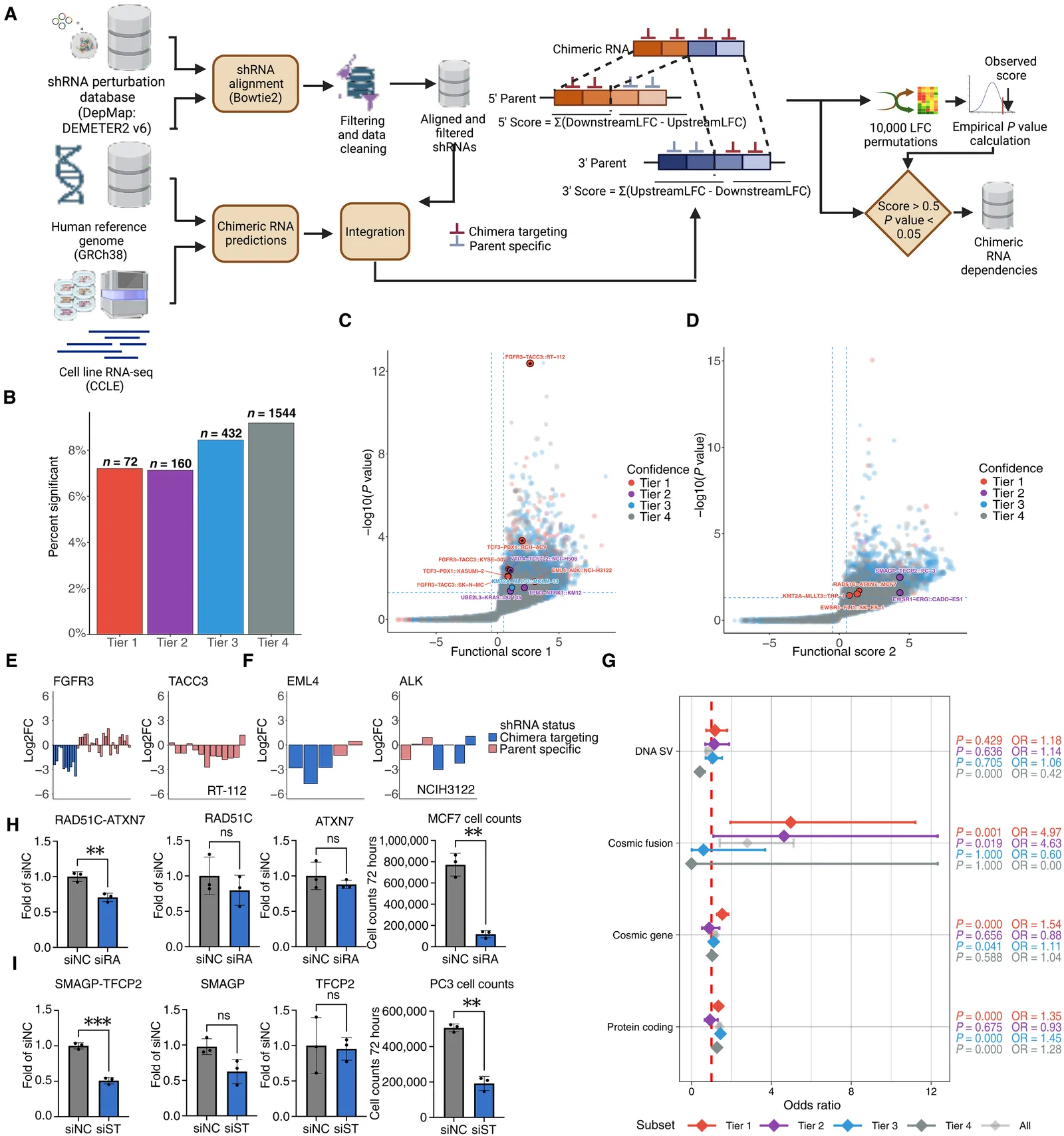
Breakpoint-aware functional dependency mapping of chimeric RNAs using RNAi. (**A**) Workflow summarizing the functional screening pipeline, including integration of RNAi knockout data, overlap of shRNAs to parental and chimeric transcripts, and calculation of functional scores. Created in BioRender. Lalani, S. (2026) https://BioRender.com/hbis8s2. (**B**) Bar graph illustrating the proportion of chimeric RNAs classified as dependencies. (**C** and **D**) Volcano plots showing functional scores for 5′ parental genes (left) and 3′ parental genes (right). COSMIC fusions are highlighted in blue passing the threshold of functional score > 0.5 and *P* value <0.05 are labeled and colored in accordance with their confidence tier, along with the two validated fusions *RAD51C-ATXN7* and *SMAGP-TFCP2*. Star denotes COSMIC fusions predicted with high confidence (*q* < 0.5). (**E** and **F**) Functional scoring profiles for representative COSMIC fusions *FGFR3-TACC3* (E) and *EML4-ALK* (F). Bars represent scaled log2-fold changes (L2FC) across the transcript from 5′ to 3′ orientation, with fusion targeting (blue) indicating shRNAs map to the chimeric transcript and parent specific (red) indicating shRNAs mapping exclusively to parental genes. Negative L2FC represents a decrease in cell viability and positive represents an increase. (**G**) Forest plot depicting enrichment of DNA structural variants, COSMIC fusion status, COSMIC parental gene status, and protein-coding potential among predicted dependent chimeric RNAs, compared to all chimeras, separated by confidence tier. Diamonds indicate odds ratios and bars represent the 95% confidence intervals. Odds ratios calculated by two-tailed Fisher’s exact test, *n* = 131,323 total chimeric RNA; chimeric RNA counts per category vary due to missing data. (**H** and **I**) Validation of select predicted functional fusions *RAD51C-ATXN7* (H) and *SMAGP1-TCFP2* (I). Leftmost panels show the expression of each chimeric RNA as measured by junction-specific qRT-PCR following siRNA-mediated knockdown. Rightmost columns show the cell counts 72 hours postknockdown. Statistical comparisons by two-tailed Welch’s *t* test (*n* = 3 per group, **P < 0.01, ***P < 0.001).

With this computational pipeline, we were able to investigate the impact of chimeric transcripts on cellular fitness across 624 cancer cell lines (fig. S3A and table S3). Of the 569,640 predicted chimeric RNAs, 131,323 chimeric RNAs (representing 30,784 unique gene pairs) were tractable in this analysis, defined as having probe coverage both upstream and downstream of the breakpoint in at least one parent. On average, each chimeric RNA was screened with 11 probes; however, because of the use of multiple dependency mapping projects in DepMap, the distribution was skewed due to Project Achilles averaging 5 probes and Project Drive averaging 20 (fig. S3B). Among screened transcripts, only 6006 chimeric RNAs were evaluable on both parental genes; the remainder were assessed via either the 5′ or 3′ parent only.

Transcripts were classified as candidate dependency events if the dependency score exceeded 0.5 and *P* value was less than 0.05 in at least one parental gene. Transcripts were further classified into “high-confidence dependency events” if they additionally passed the threshold of *q* < 0.5. Thresholds were determined by empirical observation of scores of known oncogenic fusions. This yielded 3246 candidates deemed dependent in at least one cell line with 1036 deemed high confidence. Split out into confidence tiers, we identified 72 dependencies in tier 1, 160 dependencies in tier 2, 432 dependencies in tier 3, and 1544 dependencies in tier 4 (Fig. 2B). Given the empirical nature of the scoring framework and the use of permutation-derived *P* values, these thresholds are intended to prioritize candidate dependencies rather than to define strict statistical significance.

Among the highest-scoring hits in the high-confidence category were *FGFR3-TACC3*, *TCF3-PBX1*, and *VTI1A-TCF7L2*, all well-characterized oncogenic gene fusions. Additional canonical fusions, including *EML4-ALK*, *TPM3-NTRK1*, and *EWSR1-FLI1*, also met our thresholds for candidate dependencies (Fig. 2, C to F, and fig. S4, A and B). Analysis of missed dependencies among known oncogenic fusions indicated that these cases were often attributable to lineage mismatch between the fusion’s typical indication and the screened cell line (e.g., *TMPRSS2-ERG*, a driver of prostate cancer, was only screened in the SNU-1 stomach cell line where it was not identified as a dependency) or probe design constraints (e.g., *BCL-ABL*, a hallmark of CML, was unevaluable in hematopoietic cell lines because of the absence of parent-specific probes; a low-abundance isoform in JHOS2 ovarian cells screened as nonfunctional).

To evaluate the performance of our pipeline, we conducted overrepresentation analysis within confidence tiers to assess enrichment of traits known to be associated with fusion-based dependencies among our dependent chimeric RNAs. These categories include having an oncogene or tumor suppressor gene as a parent, having a corresponding SV, and having a defined protein reading frame. We also assessed overall enrichment of known oncogenic gene fusions from the Catalogue of Somatic Mutations in Cancer (COSMIC) database (*40*), treating these as known “true positives” for fusion-based dependencies. Significant enrichment for COSMIC fusions was observed in tier 1 [odds ratio (OR) = 4.97, *P* = 0.001] and tier 2 (OR = 4.63, *P* = 0.019), while tiers 3 and 4 contained relatively few COSMIC fusions, limiting statistical power in these groups. We also observed modest but statistically robust enrichment for transcripts containing COSMIC consensus genes in tier 1 (OR = 2.71, *P* < 0.001) and tier 3 (OR = 1.43, *P* < 0.001), as well as enrichment for predicted protein-coding transcripts across tiers 1, 3, and 4 (tier 1: OR = 1.35; tier 3: OR = 1.45; tier 4: OR = 1.28; all *P* < 0.001) (Fig. 2G).

When restricting analysis to high-confidence dependency events, enrichment for COSMIC fusions was further increased in tier 1 (OR = 13.91, *P* = 0.002), but did not reach statistical significance in tier 2. Notably, several tier 2 COSMIC fusions exhibited strong functional scores but did not pass the multiple testing threshold, likely reflecting the conservative nature of the multiple testing correction rather than absence of biological relevance. Analyses of other feature categories showed patterns consistent with those observed in the broader dataset (fig. S4C). Together, these results indicate that tier 1 predictions are the most specific and enriched for classical functional dependencies derived from oncogenic gene fusions; however, biologically relevant events still exist in lower tiers.

To provide further validation for our predictions, we selected two high-scoring chimeric transcripts with high expression in common cell lines for validation, *RAD51C-ATN7* in MCF7 breast cancer cells and *SMAGP-TFCP2* in PC3 prostate cancer cell lines. We performed RNAi knockdown with siRNAs directly spanning the chimeric breakpoint and measured proliferation by cell counts. We validated the knockdown using junction-specific primers for the chimeric RNA as well as junction-specific parental gene primers to verify the specificity of the knockdown. The results of our screen showed a significant reduction in cell counts after 72 hours, supporting our predictions (Fig. 2, H and I). While we did observe a slight but nonsignificant knockdown of SMGP parent expression likely due to off target binding of our siRNA, we suspect that this does not contribute to the cell growth phenotype as SMGP is not known as a dependency in PC3 cells by DepMap CRISPR-Cas9 screening.

### Global analysis of essential cis*-*SAGe chimeric RNAs

The results of our functional screen highlighted chimeric RNAs with potential roles in cellular viability, prompting an investigation into specific classes exhibiting recurrent, biologically meaningful patterns. Cis*-*SAGe chimeric RNAs, formed through transcriptional readthrough of adjacent genes, represent a particularly compelling category as they are abundant in normal physiology, but their functional roles remain poorly characterized. Across all screened transcripts, adjacent class chimeras exhibited the strongest functional dependency signals, with significantly higher 5′ and 3′ functional scores compared to intra- and interchromosomal classes (Fig. 3, A and B). Notably, these transcripts also showed outsized recurrence across cell lines and tissues and minimal support from DNA-level SV data. Together, these features are most consistent with an RNA-level origin, such as cis-SAGe, although we note that RNA-seq alone cannot definitively establish the underlying mechanism of formation. On the basis of this enrichment and recurrence, we therefore prioritized these adjacent-class transcripts as putative cis-SAGe chimeric RNAs for further investigation.

**Fig. 3.**
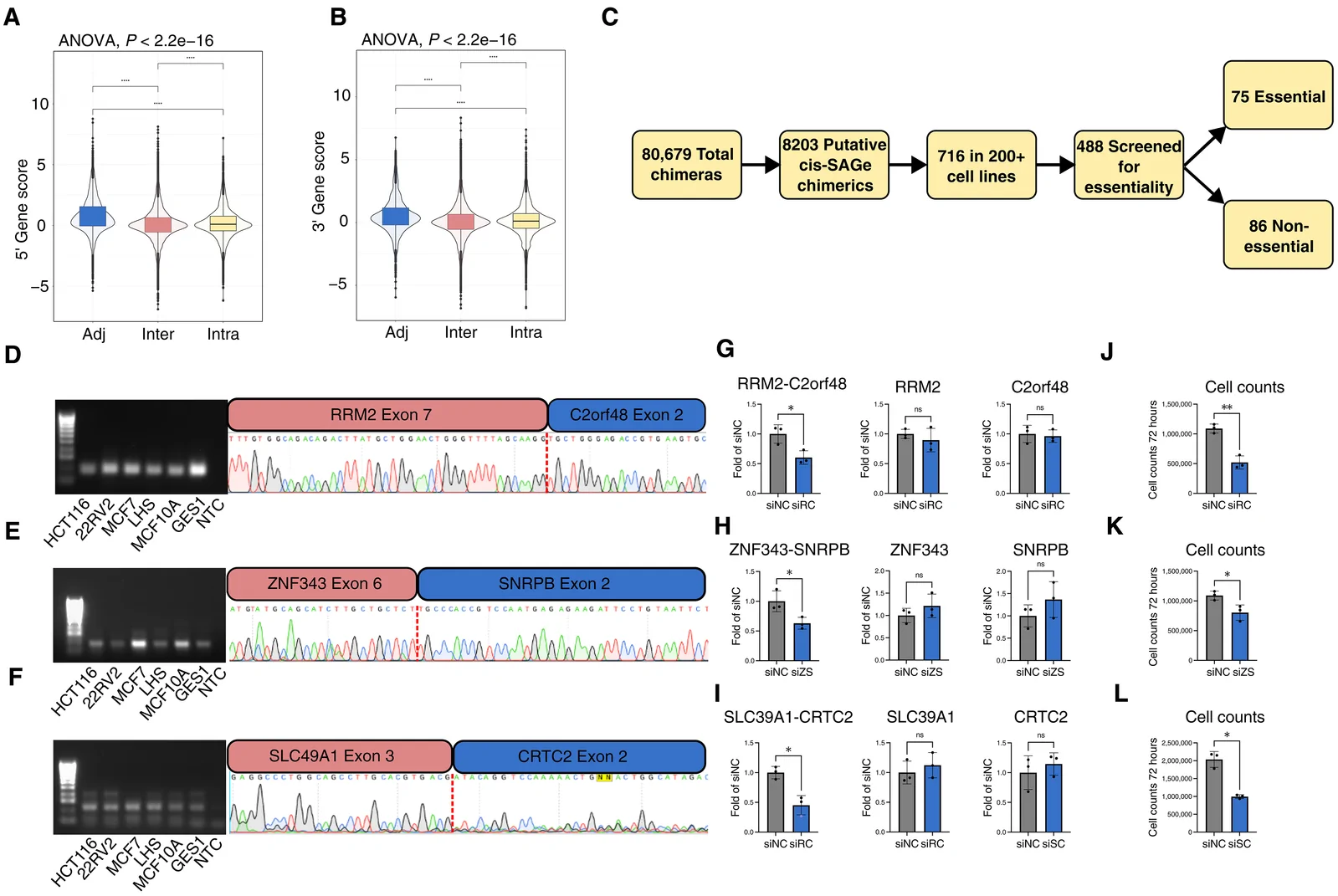
Discovery and functional validation of recurrent essential cis-SAGe chimeras. (**A** and **B**) Distributions of functional scores for the 5′ genes (A), and the 3′ gene (B). Statistical significance was assessed by one-way ANOVA followed by pairwise Welch’s *t* test adjusted for multiple comparisons by Benjamini-Hochberg correction (*n* = 4976 adjacent, 18,083 interchromosomal, and 7724 intrachromosomal, ****P < 0.0001). (**C**) Workflow detailing filtering strategy used to identify the top highly recurrent cis-SAGe chimeras classified as essential or nonessential based on functional scores derived from RNAi dependency data. (**D** to **F**) RT-PCR amplification (left) and Sanger sequencing (right) confirming the expression of three recurrent cis-SAGe chimeras: *RRM2-C2orf48* (D), *ZNF343-SNRPB* (E), and *SLC39A1-CRTC2* (F) across multiple cancer (HCT116, 22RV1, and MCF7) and noncancer (LHS, MCF10A, and GES1) cell lines with a no template control (NTC). Gel images show specific amplification of the PCR product and chromatograms display breakpoint-spanning sequences with the chimeric junction location marked in red. (**G** to **I**) qRT-PCR analysis of chimeric RNA (left), 5′ parental expression (middle), and 3′ parental expression (right) following siRNA-mediated knockdown of each chimera in HCT116 cells at 24 hours, evaluating efficacy and off target effects. Expression was normalized to GAPDH and to the nontargeting control using the ΔΔCT method. Statistical comparisons by two-tailed Welch’s *t* test (*n* = 3 per group, *P < 0.05). (**J** to **L**) Cell proliferation assays measuring total cell counts at 72 hours postknockdown for each chimera compared to nontargeting controls (siNC). Cells were initially seeded at a density of 100,000 cells per well. Statistical comparisons by two-tailed Welch’s *t* test (bars represent mean ± SD, *n* = 3 per group, *P < 0.05, **P < 0.01).

To filter for high-confidence putative cis*-*SAGe events of interest, we restricted analysis to adjacent-class chimeric RNA that were highly recurrent (*n* > 200 cell lines) and exhibited an overall median functional score > 1 in at least one parental gene across all cell lines (Fig. 3C). This strategy yielded 75 recurrent, high-confidence cis*-*SAGe transcripts (fig. S5A and table S4). To address limitations of short-read chimera detection and provide orthogonal validation, we analyzed long-read RNA-seq data from 16 ENCODE cell lines by identifying long, contiguous reads spanning the predicted chimeric breakpoints, and found that all 75 essential transcripts were supported in at least one cell line (fig. S6, A to D, and table S8). Notably, every transcript identified in this subset was also found in the GTEx dataset and none had a detectable DNA-level fusion, aligning with the hypothesis that these are RNA-level events present in varying tissue states. As a functional comparison group, we also assembled a list of nonessential cis*-*SAGe chimeric RNAs, which followed the same conditions, but had a median functional score between −0.2 and 0.2 for both parental genes, resulting in 85 nonessential (NE) transcripts (fig. S5B and table S5). Among the essential group, we detected several chimeric RNAs, namely, *RRM2-C2orf48* and *RAD51AP1-DYRK4*, previously characterized in specific cancer types (*41*–*44*), as well as *CTBS-GNG5*, a housekeeping chimeric RNA previously reported across a variety of tissues (*8*). However, the vast majority of chimeric RNAs identified have not been previously reported outside of broad landscaping studies, underscoring the breadth of our dataset. As expected, the essential group exhibited higher functional scores than the nonessential groups (fig. S8, A and B); however, we did not observe a difference in expression levels (fig. S8C). We did observe an increase in the proportion of transcripts with defined protein reading frames (fig. S8D) and transcripts exhibiting canonical splicing (fig. S8D). This observation suggests that essential chimeric RNAs may use canonical splicing mechanisms to generate noncanonical RNA transcripts and fusion proteins.

To investigate the predicted cis*-*SAGe chimeric RNAs in vitro, we selected three identified essential transcripts: *RRM2-C2orf48*, *ZNF343-SNRPB*, and *SLC39A1-CRTC2*. While *RRM2-C2orf48* has been reported in specific cancers (*41*–*43*), its function as a recurrent essential transcript has not been explored. *ZNF343-SNRPB* and *SLC39A1-CRTC2* have been detected in landscaping studies but neither have received functional characterization to date (*6*, *45*). For each transcript, we designed PCR primers specifically amplifying the chimeric junction and confirmed expression across a panel of cancerous (HCT116: large intestine, 22RV1: prostate, and MCF7: breast) and noncancerous (LHS: prostate, MCF10A: breast, and GES1: stomach) cell lines encompassing a variety of tissues (Fig. 3, D to F). On-target amplification was confirmed by Sanger sequencing in the HCT116 cells and alignment to the predicted chimeric junction region. Similarly, we also amplified and sequenced additional transcripts in HCT116 cells to demonstrate the accuracy of our predictions (fig. S7, A to E). Our results revealed that these transcripts were expressed in all cell lines screened, suggesting a broad role in cellular physiology.

To confirm the role of the essential transcripts in cell proliferation, we designed siRNAs spanning the chimeric junction to specifically knock down three transcripts (*RRM2-C2orf48*, *ZNF343-SNRPB*, and *SLC39A1-CRTC2*) without affecting the parental genes. We transfected these probes into HCT116 cells and measured the expression of the target chimeric RNA, as well as the parental gene expression after 24 hours by qRT-PCR (Fig. 3, G to I), observing a significant knockdown of all chimeric transcripts with no significant change of expression of the parental genes. Knockdown for all three transcripts resulted in a significant decrease in cell counts at 72 hours, in agreement with our functional screen predictions and supporting a role in cell viability (Fig. 3, J to L).

### Characterization of pathways associated with essential cis*-*SAGe chimeric RNA expression

We next sought to identify relevant biomolecular pathways associated with cis*-*SAGe chimeric RNAs. To investigate this bioinformatically, we stratified cell lines as high versus low chimeric RNA expression by median absolute deviation (MAD) outlier analysis. Differentially expressed genes (DEGs) between the groups were identified using DESeq2, followed by gene set enrichment analysis (GSEA) against the MSigDB Hallmarks collection (*46*, *47*) to identify pathways enriched in cell lines with high expression of each identified chimeric RNA (Fig. 4A and table S6). We observed an enrichment of cell cycle–associated gene sets among a large proportion of the chimeric RNAs, specifically E2F Targets, MYC Targets V1, and G2M Checkpoint. As a control, we performed the same analysis for the nonessential cis*-*SAGe chimeric RNAs (table S7). A direct comparison between the two datasets by the Fisher’s exact test confirmed that cell cycle gene sets were more frequently enriched in the essential group compared to the nonessential group (Fig. 4B). Specifically, the E2F Targets and MYC Targets V1 showed significantly higher enrichment scores in the essential cis*-*SAGe chimeras compared to the nonessential controls by following FDR correction (adj_*P* < 0.05). These associations suggest that expression of many cis*-*SAGe chimeric RNAs occurs in cellular contexts characterized by increased proliferation and cell cycle activity.

**Fig. 4.**
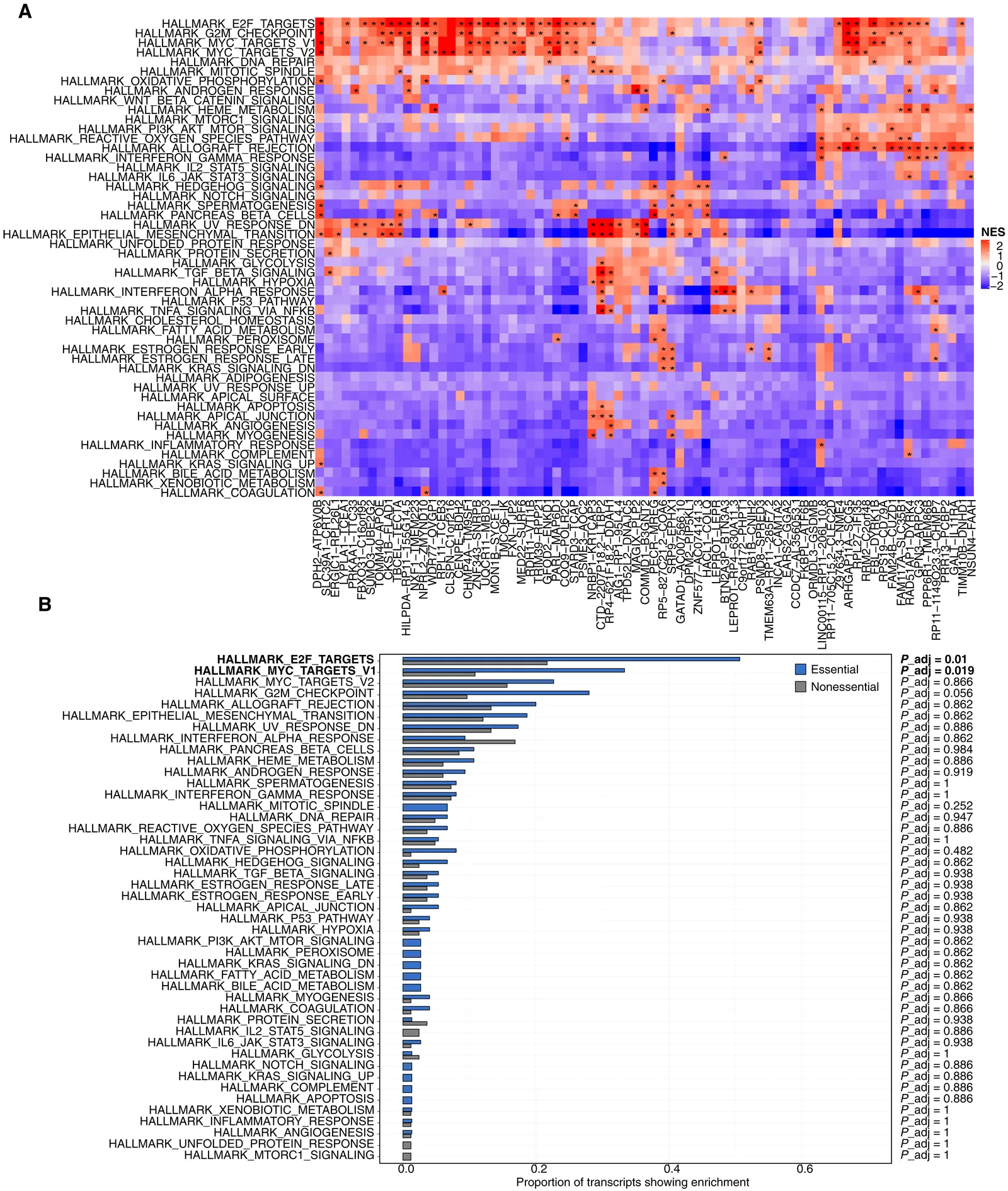
Functional enrichment analysis of essential cis-SAGe chimeric RNAs. (**A**) Heatmap of normalized enrichment scores (NES) from GSEA comparing cell lines with high expression of each essential chimeric RNA to all others as determined by median absolute deviation. Red indicates positive NES and blue indicates negative NES, reflecting pathway enrichment or depletion. Dots signify significantly enriched pathways (*q* value <0.05 and NES > 1). (**B**) Comparison of the proportion of transcripts exhibiting significant pathway enrichment between essential (blue) and nonessential (gray) cis-SAGe chimeras. *P* values were calculated by two-tailed Fisher’s exact test and adjusted by Benjamini-Hochberg correction for multiple testing. Bold pathways indicate adj_*P* < 0.05.

To experimentally assess the functional consequences of select transcripts, we investigated the perturbation of *RRM2-C2orf48* and *ZNF343-SNRPB* as both showed significant enrichments of cell cycle–linked pathways MYC targets and E2F targets in the pathway enrichment analysis (fig. S9, A to D). First, we knocked down *RRM2-C2orf48* in HCT116 cells and performed RNA-seq analysis, which showed suppression of the same MYC targets and E2F targets pathways (fig. S9E). KEGG pathway analysis of the RNA-seq data further revealed an activated p53 response, marked by transcriptional up-regulation of the downstream target p21 and down-regulation of E2F target genes (fig. S9F). Next, we measured cell cycle distribution following perturbation of each chimeric RNA using flow cytometry with 4′,6-diamidino-2-phenylindole (DAPI) to measure DNA content and EdU to identify cells actively undergoing nascent DNA synthesis. This permitted us to quantify cell cycle disruption at various stages: G_1_, S, or G_2_-M. We observed an accumulation of cells in G_1_ phase with the knockdown of *RRM2-C2orf48* and an accumulation of cells in the G_2_-M phase with the knockdown of *ZNF343-SNRPB* (Fig. 5A and fig. S10, A to D). These results indicate that loss of these individual chimeric transcripts disrupts normal cell cycle progression, consistent with impaired cellular fitness and proliferative capacity providing experimental evidence for essentiality.

**Fig. 5.**
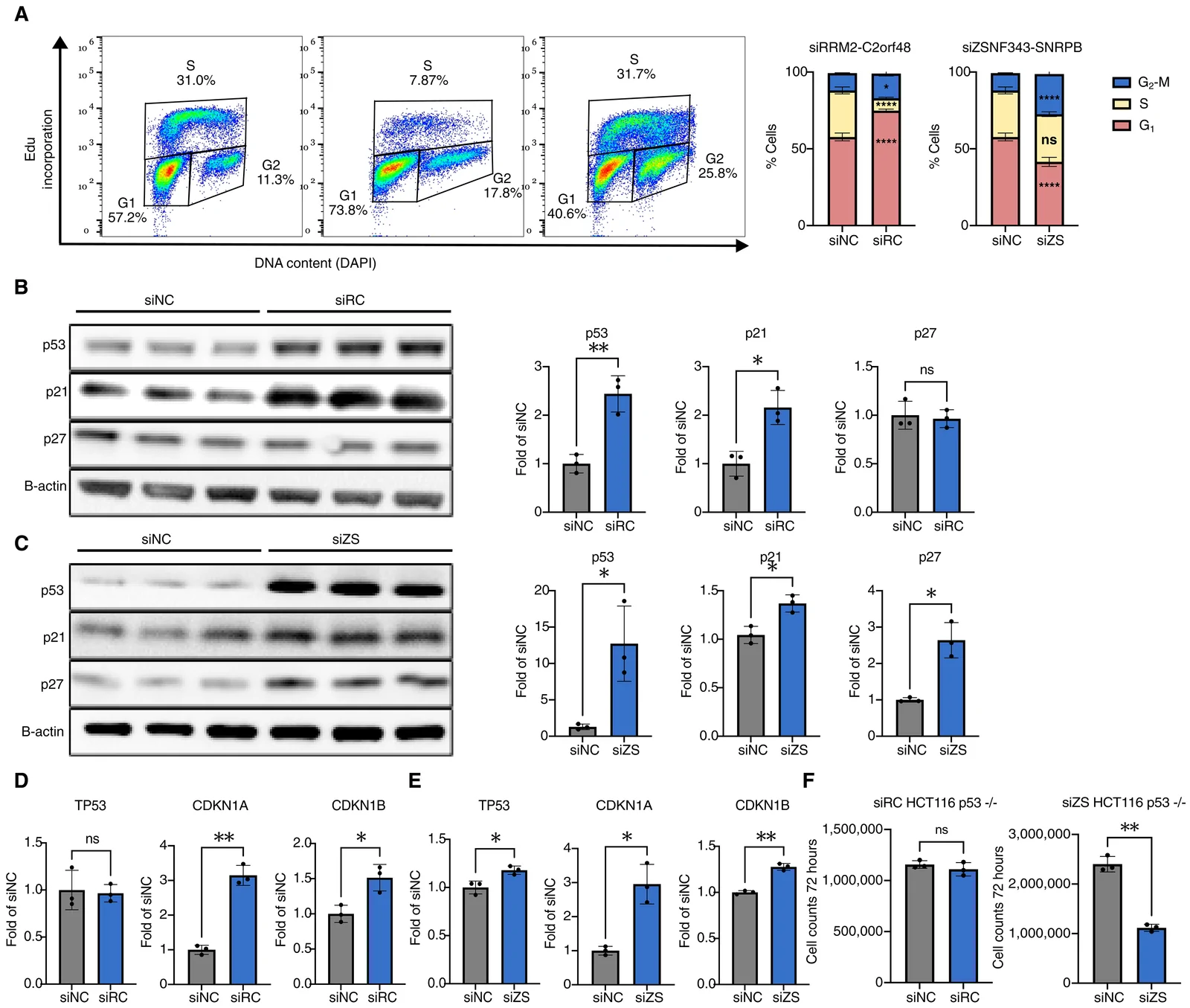
Functional analysis of select essential cis-SAGe chimeric RNAs. (**A**) Flow cytometry analysis of cell cycle distribution using EdU incorporation and DAPI staining at 72 hours postknockdown in HCT116 cells. Representative plots for siNC and each chimera-specific siRNA (*RRM2-C2orf48*, *ZNF343-SNRPB* left to right) are shown (all plots are shown in fig. S10). EdU incorporation (nascent DNA synthesis/S-phase) is presented on the *y* axis and DAPI (relative DNA content) is presented on the *x* axis. Quantification is shown on the right. Statistical significance was assessed using two-way ANOVA with Sidak’s multiple testing comparison (bars represent mean ± SD, *n* = 3 replicates per group, *P < 0.05, ****P < 0.0001). (**B** and **C**) Western blot analysis of p21, p27, and p53 following siRNA-mediated knockdown of *RRM2-C2orf48* (B) and *ZNF343-SNRPB* (C) in HCT116 cells at 72 hours. Left panels show representative band images, and right panels shows quantitation of band intensity normalized to B-actin. Statistical significance was assessed using Welch’s *t* test (bars represent mean ± SD, *n* = 3 replicates per group, *P < 0.05, **P < 0.01). (**D** and **E**) qRT-PCR analysis of gene expression changes of TP53 and downstream targets *CDKN1A* and *CDKN1B* following siRNA-mediated knockdown of each chimera in HCT116 cells at 72 hours. Expression was normalized to GAPDH and to the nontargeting control using the ΔΔCT method. Statistical significance was assessed using Welch’s *t* test (bars represent mean ± SD, *n* = 3 replicates per group, *P < 0.05, **P < 0.01). (**F**) Cell proliferation assays in p53-null HCT116 cells measuring total cell counts at 72 hours postknockdown for *RRM2-C2orf48* (left) and *ZNF343-SNRPB* (right) compared to nontargeting controls (siNC). Cells were initially seeded at a density of 100,000 cells per well. Statistical comparisons by two-tailed Welch’s *t* test (bars represent mean ± SD, *n* = 3 per group, **P < 0.01).

Given the observed cell cycle impairment following perturbation of essential cis*-*SAGe, we next asked whether these effects were mediated through activation of canonical cell cycle checkpoint pathways. Because p53 is the central regulator of stress-induced cell cycle arrest (*48*), we hypothesized that perturbation of essential cis*-*SAGe chimeras would activate a p53-dependent checkpoint response. Consistent with this model, siRNA-mediated knockdown of either *RRM2-C2orf48* or *ZNF343-SNRPB* in HCT116 cells led to stabilization of p53 protein across both chimeras, accompanied by increased protein levels of p53’s downstream effector p21 in *RRM2-C2orf48* and *ZNF343-SNRPB* knockdown conditions with an increase in p27 as well in the *ZNF343-SNRPB* knockdown (Fig. 5, B and C). Similarly, this p53 activation was observed at the transcriptional level, with subsequent significant transcriptional up-regulation of downstream targets *CDKN1A* (p21) and *CDKN1B* (p27) following both knockdowns. *TP53* mRNA expression levels were unchanged in the *RRM2-C2orf48* knockdown and only showed a modest increase in the *ZNF343-SNRPB* knockdown, suggesting a primarily posttranscriptional mechanism of regulation (Fig. 5, D and E). Knockdown of these transcripts in HCT116 p53-null cells revealed differential effects. In the case of *RRM2-C2orf48*, loss of the chimeric RNA rescued the cell growth impairment observed in WT cells, supporting a model in which p53-dependent checkpoint activation mediates the phenotype. In contrast, the growth defect associated with *ZNF343-SNRPB* depletion persisted in p53-null cells, indicating that additional p53-independent mechanisms contribute to its functional role (Fig. 5F).

Together, these data indicate that perturbation of selected cis*-*SAGe chimeric RNAs induces cell cycle disruption and activation of checkpoint-associated responses. However, we note that these effects may arise through diverse upstream mechanisms, and the extent to which individual chimeras directly regulate cell cycle pathways remains to be determined. Notably, similar proliferative phenotypes arising from depletion of distinct chimeras suggest that these transcripts may affect cellular fitness through diverse molecular mechanisms that converge on cell cycle disruption.

### Mechanistic classification of essential cis-SAGe chimeric RNAs

To investigate the mechanisms underlying essential cis-SAGe chimeric RNAs, we performed integrative characterization of predicted essential candidates across protein, transcript, and localization features. Our predicted cis*-*SAGe chimeric RNAs exhibit diverse transcript structures. To illustrate this, we examined transcript architecture and open reading frames (ORFs) for three representative, previously validated transcripts. While all three use canonical start sites derived from their 5′ gene, only *SLC39A1-CRTC2* continues into the coding region of the 3′ gene, generating an in-frame fusion product. In contrast, *ZNF343-SNRPB* introduces a frameshift leading to premature termination within an exon, while *RRM2-C2orf48* produces an in-frame transcript that extends into an unannotated region of *C2orf48* lacking a defined coding sequence (Fig. 6A).

**Fig. 6.**
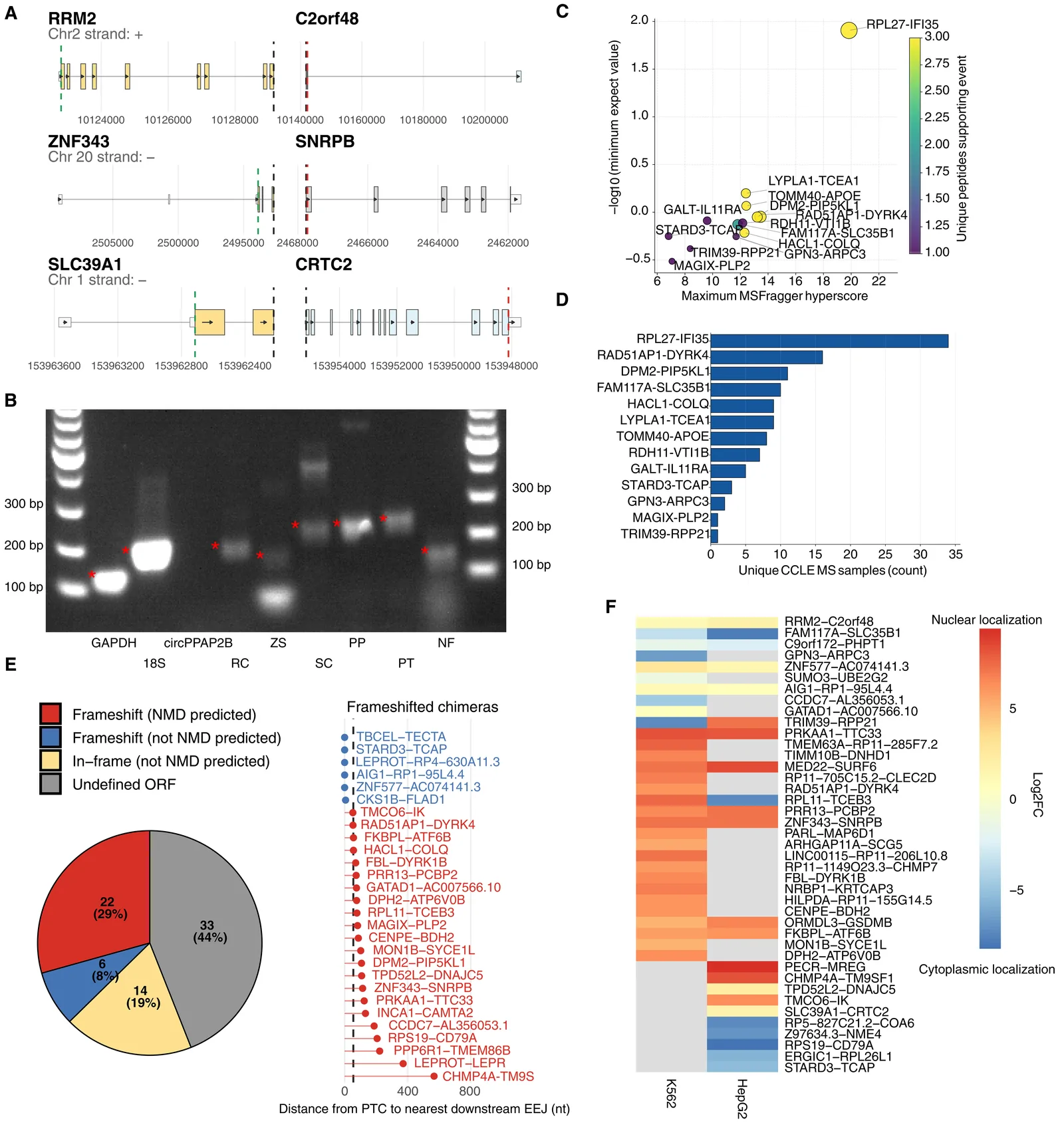
Mechanistic classification of essential cis-SAGe chimeric RNAs. (**A**) Transcript and ORF diagrams of the essential cis-SAGe chimeric RNAS *RRM2-C2orf48*, *ZNF343-SNRPB*, and *SLC39A1-CRTC2*. Large boxes represent coding regions (CDSs) and small boxes represent untranslated regions (UTRs). Dashes represent start codons (green), stop codons (red), and chimeric breakpoints (gray). Transcripts are shown in the 5′ to 3′ orientation. (**B**) RT-PCR analysis of ribosome pulldown products targeting the chimeric junction sequence. 18*S* serves as the pulldown control, *GAPDH* serves as a positive control for coding transcripts, and *circPPAP2B* serves as a negative control for noncoding transcripts. Red asterisks indicate bands matching the expected size (Fig. 3, D to F, and fig. S7, A to C). (**C**) Bubble plot showing mass spectrometry support for predicted fusion peptides across 392 CCLE mass spectrometry runs. The *x* axis indicates maximum MSFragger hyperscore and the *y* axis indicates analytical significance (−log10 minimum expectation value). Bubble size represents the number of peptide-spectrum matches (PSMs), and color denotes the number of unique supporting peptides. (**D**) Horizontal bar chart showing the distribution of fusion peptides across the cohort. Peptides are ordered by the number of unique cell lines containing at least one positive PSM. (**E**) Nonsense-mediated decay (NMD) predictions for essential cis-SAGe chimeric RNAs. The pie chart (left) shows the distribution of transcript classifications, and the lollipop plot (right) shows the distance between the frameshift-induced premature termination codon (PTC) and the nearest downstream exon-exon junction (EEJ) for all predicted frameshifted transcripts. (**F**) Subcellular localization of chimeric RNAs in K562 and HepG2 cells. Log2 fold change was calculated from the mean fusion fragments per million (FFPM) across replicates, comparing nuclear and cytoplasmic fractions. Positive values (red) indicate nuclear enrichment, whereas negative values (blue) indicate cytoplasmic enrichment. Gray denotes chimeric RNAs not detected in either fraction.

Because transcript structure alone does not establish whether these chimeras engage the translational machinery, we next performed ribosome pulldown assays to analyze ribosome occupancy. Out of the six transcripts we screened, all amplified within ribosome-associated RNA fractions (Fig. 6B), demonstrating that these transcripts can associate with ribosomes and are available for translation. However, ribosome association alone does not establish productive protein synthesis, as many noncoding transcripts and regulatory RNAs can also interact with ribosomes. Therefore, we performed additional analyses to determine whether essential cis-SAGe transcripts exhibit evidence of stable protein production or are more consistent with RNA-mediated mechanisms.

To assess additional evidence of protein translation among the larger dataset, we generated predicted peptide sequences from all defined coding regions, performed in silico tryptic digestion, and filtered for junction-specific peptides absent from the canonical proteome, leaving junction spanning and frameshift-derived peptides. These peptides were queried against mass spectrometry data from 392 CCLE cancer cell line runs using MSFragger (Fig. 6, C and D, and table S9) (*49*, *50*). Because of constraints in peptide detectability, specifically the requirement for peptides spanning fusion junctions or derived from downstream frameshifts, only 23 chimeras were evaluable; nonetheless, peptide-spectrum matches (PSMs) were identified for 13 of these, providing direct evidence of translation for a subset of chimeric RNAs. The transcripts with the most proteomic support include *RPL27-IFI35* (total PSMs = 39, max hyperscore = 19.843, and min expected value = 0.0124) *RAD51AP1-DYRK4* (total PSMs = 16, max hyperscore = 13.454, and min expected value = 0.1172), and *DPM2-PIP5KLI* (total PSMs = 12, max hyperscore = 13.22, and min expected value = 1.1288). While these hyperscores and expected values are modest in the context of traditional proteomics, we nonetheless detect reproducible peptide-spectrum evidence supporting translation, suggesting that fusion-derived peptides are present but remain challenging to capture with standard proteomic approaches.

Despite this evidence of translation for some candidates, many chimeric RNAs were predicted to encode frameshifted transcripts, which are known to induce premature termination codons (PTCs) and subject the transcript to nonsense-mediated decay (NMD). To determine whether this is the case for our essential cis-SAGe chimeras, we identified and measured frameshift-induced PTCs relative to downstream exon-exon junctions (EEJs), classifying transcripts as NMD substrates if the PTC is exceeding 50 nt of the next downstream EEJ. The majority of frameshifted transcripts (22 of 28) met criteria consistent with NMD targeting (Fig. 6E and table S10), suggesting that a substantial fraction of frameshifted essential chimeric RNAs do not function through stable protein products.

To further distinguish potential modes of action, we analyzed subcellular localization using RNA-seq data from nuclear and cytosolic fractions in ENCODE cell lines (*51*). Applying our quantification pipeline, we observed both nuclear and cytoplasmic enrichment across detected chimeric RNAs. Notably, localization patterns were concordant with coding potential: Chimeric RNAs with proteomic support, such as *STARD3-TCAP* and *FAM117A-SLC35B1*, were enriched in the cytoplasm, consistent with protein-coding function. In contrast, transcripts predicted to undergo NMD, including *ZNF343-SNRPB* and *FKBPL-ATF6B*, were preferentially localized to the nucleus, suggesting potential noncoding regulatory roles (Fig. 6F and table S11).

One notable discrepancy was *RAD51AP1-DYRK4*, which showed strong proteomic evidence in the full cell line dataset but was predominantly nuclear at the transcript level in K562 cells and predicted to undergo NMD. *RAD51AP1-DYRK4* has previously been shown to encode a stable oncogenic fusion protein in luminal B breast cancer, where it drives MEK/ERK signaling (*44*). We speculate that in certain tumor microenvironments where NMD activity is suppressed, such as hypoxic conditions (*52*), sufficient cytoplasmic transcript may accumulate to permit translation of the fusion protein and drive oncogenic signaling. Whether the nuclear transcript additionally functions as a regulatory nuclear RNA in this or other cellular contexts remains to be determined.

Together, these results demonstrate that essential cis*-*SAGe chimeric RNAs are mechanistically heterogeneous, comprising both protein-coding fusion transcripts and noncoding RNAs with likely regulatory functions.

### Characterization of essential cis*-*SAGe chimeric RNAs in breast cancer

Transcriptional dysregulation is increasingly recognized as a staple phenotype of cancer, and intergenic splicing has emerged as an underexplored contributor to transcriptomic diversity in tumors (*53*, *54*). To investigate the role of cis*-*SAGe events in cancer biology, we applied our prediction pipeline to RNA-seq data from 1059 primary breast tumors and 95 tumor-adjacent normal tissues from The Cancer Genome Atlas (TCGA) breast cancer cohort (*55*) (Fig. 7A and table S12). This analysis yielded 36,482 total unique chimeric RNA predictions with 7933 (21.7%) adjacent class chimeric RNAs.

**Fig. 7.**
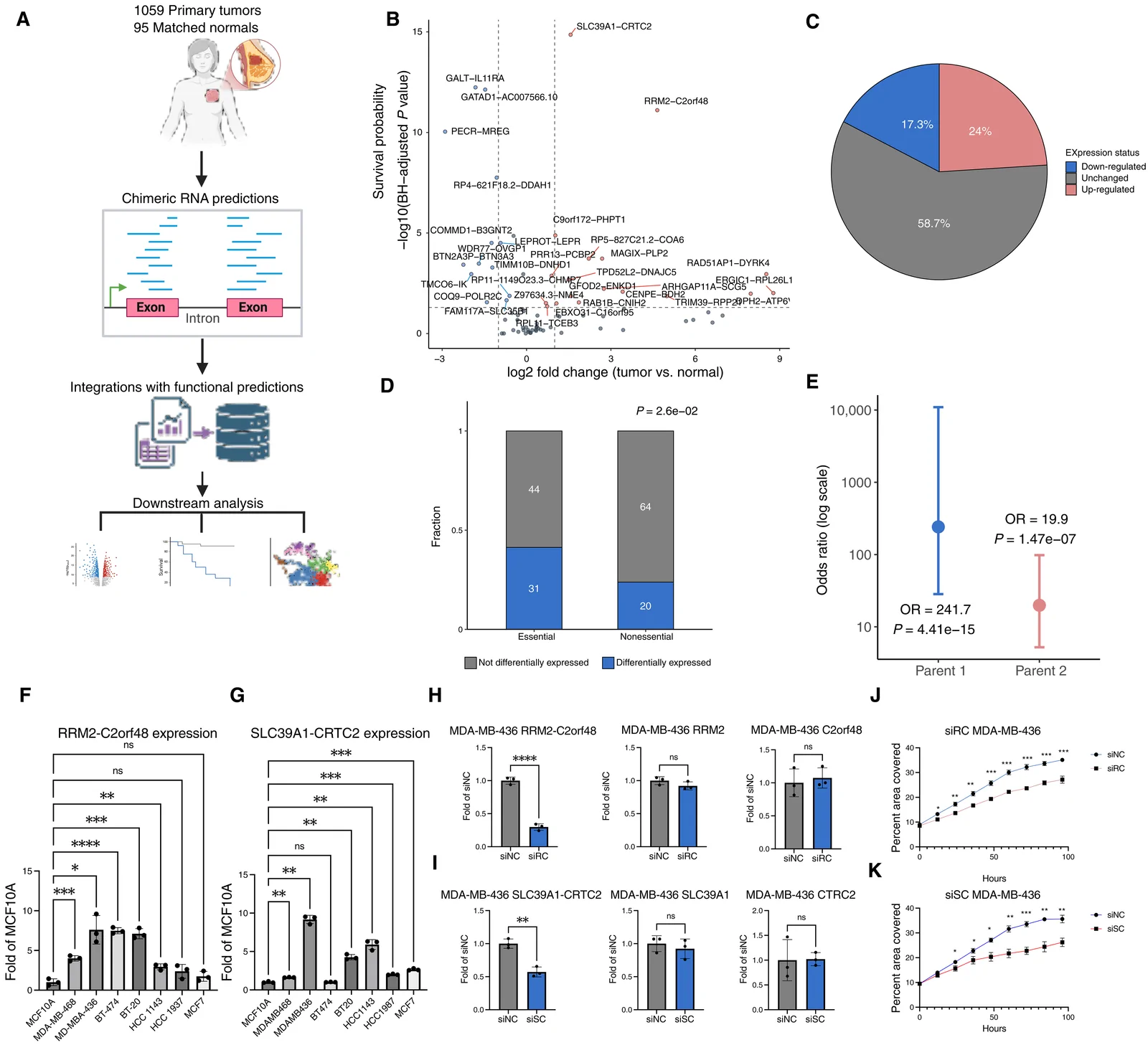
Translational impact of essential cis-SAGe chimeric RNAs in breast cancer. (**A**) Workflow for patient sample analysis. Created in BioRender. Lalani, S. (2026) https://BioRender.com/cn5buza. (**B**) Differential expression of essential cis-SAGe chimeras in tumor versus normal breast tissue (Wilcoxon rank-sum test, Benjamini-Hochberg correction). Red, up-regulated in tumor (log_2_FC > 1, FDR < 0.05); blue, down-regulated (log_2_FC < −1, FDR < 0.05). (**C**) Summary of tumor-versus-normal expression changes for essential chimeras. (**D**) Comparison of differential expression between essential and nonessential chimeras (two-way Fisher’s exact test, *P* = 0.026). (**E**) Fisher’s exact test for co-occurrence of differential expression between essential chimeras and their 5′ and 3′ parental genes (error bars: 95% CI; 5′ parent: OR = 241.7, *P* = 4.4 × 10^*−*15^; 3′ parent: OR = 19.9, *P* = 1.5 × 10^*−*7^). (**F** and **G**) qRT-PCR validation of *RRM2-C2orf48* and *SLC39A1-CRTC2* across breast cancer cell lines and normal control (Brown-Forsythe test, *P* < 0.0001; pairwise Welch’s *t* test; mean ± SD, *n* = 3; *P < 0.05, **P < 0.01, ***P < 0.001, ****P < 0.0001). (**H** and **I**) qRT-PCR of chimeric, 5′ parental, and 3′ parental expression after siRNA knockdown of *RRM2-C2orf48* and *SLC39A1-CRTC2* in MDA-MB-436 cells at 24 hours (Welch’s *t* test; mean ± SD, *n* = 3; **P < 0.01, ****P < 0.0001). (**J** and **K**) Cell proliferation assays after siRNA knockdown of *RRM2-C2orf48* and *SLC39A1-CRTC2* in MDA-MB-436 cells (Welch’s *t* test; mean ± SD, *n* = 3 per group/time point; *P < 0.05, **P < 0.01, ***P < 0.001).

We assessed the potential of cis*-*SAGe chimeric RNAs as prognostic biomarkers by quantifying the number of unique adjacent transcripts to estimate the cis*-*SAGe burden for each patient (table S13). We then stratified them into quartiles and compared overall survival using Kaplan-Meier curves and a log rank test, which revealed a trend toward shorter overall survival in patients with high cis*-*SAGe burden (Q3) compared to those with low cis*-*SAGe burden (Q1) (fig. S11A, *P* = 0.055, HR = 1.9). Although the survival association did not reach conventional significance (*P* < 0.05), the hazard ratio of 1.9 suggests a potentially meaningful clinical effect. Next, we used a univariate Cox proportional hazards model to assess the prognostic potential of individual chimeric RNAs, using expression (FFPM) as a continuous covariate. Several transcripts correlated with survival, with *FAM24B-CUZD1* and *NRBP1-KRTCAP3* remaining statistically significant after FDR correction (fig. S11B and table S14). These results highlight specific chimeric RNAs as potential independent biomarkers for breast cancer prognosis.

We then turned our attention to our essential cis*-*SAGe transcripts, where all transcripts were detected in the breast cancer cohort. To evaluate subtype-specific expression, we calculated the Tau specificity score for each essential cis*-*SAGe chimeric RNA identified in our screen (fig. S11C and table S15). This analysis revealed a spectrum of subtype-biased expression, with *ORMDL3-GSDMB* exhibiting the highest specificity for the HER2-enriched subtype, followed by *CLSPN-C1orf216* (Luminal B) and *CTD-2207P18.2-LTBP2* (Basal), all of which exceeded the established 0.8 threshold for tissue specificity. However, the majority of transcripts fell below the cutoff for high specificity, signaling expression across various molecular environments, consistent with our cell line analysis.

We then asked whether these essential cis*-*SAGe chimeric RNAs are dysregulated in breast cancer. Using gene expression values from our quantitation pipeline (in FFPM), we calculated log_2_FCs for each essential cis*-*SAGe transcript between tumor and adjacent normal tissue and evaluated statistical significance using the Wilcoxon rank-sum test with Benjamini-Hochberg correction. Transcripts with log_2_FC > 0.6 and adjusted *P* < 0.05 were classified as up-regulated, while those with log_2_FC < −0.6 and adjusted *P* < 0.05 were classified as down-regulated (Fig. 7B). Overall, 41% of essential cis*-*SAGe chimeric RNAs were deemed to be differentially expressed with 24% up-regulated and 17.3% down-regulated (Fig. 7C and table S16). This proportion was significantly higher than that observed for nonessential chimeric RNAs (23.8%; Fisher’s exact *P* = 0.026) (Fig. 7D). Notably, we observed concordant dysregulation between chimeric transcripts and their parental genes, consistent with shared regulatory influences. However, this concordance was not uniform across both parental loci: Chimeric RNA expression more closely mirrored that of the 5′ parental gene than the 3′ gene, suggesting that transcriptional regulation of these chimeras may be predominantly driven by the 5′ promoter (Fig. 7E).

The previously characterized transcripts *RRM2-C2orf48* and *SLC39A1-CRTC2* were among the differentially expressed chimeric RNAs highlighting their importance across various cell and disease states. We measured expression by qRT-PCR across a panel of breast cancer cell lines (MDA-MB-436, MDA-MB-468, BT-474, BT-20, HCC1143, HCC1937, and MCF7) and the noncancerous breast epithelial cell line MCF10A. The resulting expression patterns mirrored the patient data, with *RRM2-C2orf48* showing significant up-regulation in five of seven cell lines and *SLC39A1-CRTC2* showing significant up-regulation in six of seven cell lines (Fig. 7, F and G). Furthermore, knockdown of each transcript in the breast cancer cell line MDA-MB-436 showed a significant decrease in proliferation over time, highlighting their importance in cancer cell proliferation (Fig. 7, H to K).

## DISCUSSION

Our overall findings position chimeric RNAs as a substantial and functionally relevant component of the human transcriptome. By integrating a large-scale atlas of chimeric RNAs with transcriptome-wide RNAi functional data, we conducted a systematic functional analysis and identified a landscape of essential cis-SAGe transcripts that exhibit reproducible functional signals across diverse cellular lineages, supporting the idea that these transcripts participate in core cellular processes rather than representing incidental by-products of transcription. In addition, our study demonstrates that transcript-level functional dependencies can be estimated from legacy RNAi-based perturbation datasets, providing a strategy for characterizing biologically relevant noncanonical transcripts.

In our study, we assembled a set of 75 cis-SAGe chimeric RNAs that exhibit widespread functional dependencies and expression. We successfully recapitulated several previously described highly recurrent cis-SAGe chimeras, including *CTNNBIP1-CLSTN1* and *CTBS-GNG5* (*8*, *42*, *56*, *57*), while also identifying numerous previously undescribed transcripts. We further performed functional characterization of two previously unprofiled transcripts, *ZNF343-SNRPB* and *SLC39A1-CRTC2*, and expanded our understanding of *RRM2-C2orf48* by examining its expression and essentiality across tissues and cellular states (*41*–*43*). Notably, the transcripts emerging from our dependency analyses were frequently associated with cell cycle–related gene expression programs, and perturbation of representative candidates impaired proliferation and disrupted cell cycle progression. While the specific molecular mechanisms remain incompletely understood, these findings suggest that functional cis-SAGe transcripts are integrated into cellular pathways that influence fitness and proliferative capacity rather than representing incidental products of intergenic splicing. Together, these observations support a broader role for intergenic splicing in shaping the functional transcriptome than has traditionally been appreciated.

Our mechanistic analyses further suggest that essential cis-SAGe chimeric RNAs cannot be viewed as a single functional class. While several transcripts showed proteomic and ribosomal evidence consistent with translation, many others were predicted to undergo NMD and exhibited preferential nuclear localization. These observations support a model in which cis-SAGe transcripts contribute to cellular fitness through both protein-coding and noncoding mechanisms. Furthermore, our observations surrounding *RAD51AP1-DYRK4* suggest that the distinction between coding and noncoding chimeric RNAs may not always be binary. Previous studies have shown that chimeric RNAs can exert biological effects independent of their encoded proteins, as demonstrated for *SLC45A3-ELK4* (*10*), while others such as *ERCC1-iASPP* (*56*) have been suggested to exhibit both protein-coding and RNA-mediated activities. Therefore, it remains possible that some chimeric transcripts can occupy an intermediate functional state in which both the transcript and encoded protein contribute to biological function. This extends the traditional view of chimeric RNAs and suggests that intergenic splicing can generate a diverse repertoire of functional RNA molecules with distinct molecular modes of action.

While investigating the clinical impact of chimeric RNAs, we demonstrate that cis*-*SAGe chimeric RNAs play a pivotal role in breast cancer physiology. Many of our predicted essential cis*-*SAGe transcripts were found to be dysregulated in patient samples compared to adjacent normal tissue, suggesting a cancer-specific response. The highly significant up-regulation of *SLC39A1-CRTC2* is of particular interest, as previous studies have linked *CRTC2* expression to progression of non–small cell lung cancer, ovarian cancer, and head and neck squamous cell carcinoma (*57*–*59*). Our screen identified this transcript as forming an in-frame fusion protein possibly containing a functional portion of the *CRTC2* protein. A previous study that investigated fusion proteins derived from chimeric RNAs detected the fusion junction peptide by mass spectrometry, validating its coding prediction and suggesting a potential mechanism for *CRTC2* dysregulation in cancer (*6*).

Looking forward, a critical area of investigation involves elucidating the upstream molecular mechanisms that govern the biogenesis of cis*-*SAGe chimeric RNAs. While our study focuses on the functional consequences and dependencies associated with these transcripts, the regulatory landscape that facilitates read-through transcription and subsequent intergenic splicing remains largely undefined. Emerging evidence suggests that cis*-*SAGe events are intricately linked to the chromatin architecture of the parental loci (*60*). Future studies integrating epigenetic profiling methods like ChIP-seq and HI-C with chimeric RNA profiles will be essential to determine how the epigenetic landscape dictates intergenic splicing patterns. This phenomenon of cis*-*spliced chimeric RNAs closely resembles downstream-of-gene (DoG) RNAs, transcripts that extend beyond canonical transcriptional termination sites, often in response to cellular stress or compromised transcriptional termination machinery (*61*). It remains to be determined whether the functional cis*-*SAGe chimeras identified in our screen represent a specialized subset of DoG RNAs that have acquired stable splicing patterns, or if they constitute a distinct class of regulated transcripts. Deciphering these mechanisms may ultimately reveal whether modulating chromatin modifiers or termination factors can serve as a viable therapeutic strategy to suppress the production of oncogenic read-through events.

A key limitation of this study is that functional data were derived exclusively from cancer cell lines, as transcriptome-scale dependency screens are currently available only in malignant models. To mitigate this bias, we focused on chimeric RNAs expressed across diverse lineages and compared our predictions with GTEx normal tissues, where widespread expression of essential cis*-*SAGe transcripts suggests conserved biological roles rather than cancer-specific artifacts. Another limitation is the design of the DepMap RNAi libraries, which were optimized for gene-level essentiality rather than transcript-segment resolution. Inadequate probe coverage around some fusion junctions likely caused certain functional effects to be underestimated or missed. Thus, the functional candidates identified here represent a high-confidence subset rather than an exhaustive catalog. In addition, RNAi-based dependency datasets are subject to off-target effects and variable knockdown efficiency, which can introduce false-positive or inconsistent gene essentiality signals. To mitigate these effects, we focused on highly recurrent dependencies observed across large numbers of cell lines and required multiple independent perturbations targeting each side of the breakpoint to avoid signals being driven by a single shRNA, thereby reducing the likelihood that individual off-target effects drive the observed associations. Future studies incorporating junction-targeted libraries or CRISPR-Cas13 approaches with improved transcript-level specificity will enable more comprehensive discovery of functional chimeric RNAs.

In conclusion, our study provides analysis of the biological prevalence and functional relevance of chimeric RNAs. By integrating an expansive transcriptomic atlas with large-scale dependency screens, we have demonstrated that chimeric RNAs, including those arising from cis*-*SAGe events, are not merely transcriptional by-products but are essential regulators of cellular physiology and potential drivers of malignancy. Our findings in breast cancer further underscore the clinical utility of these transcripts as both prognostic biomarkers and potential therapeutic vulnerabilities. As we move toward a more complete understanding of the noncanonical transcriptome, the transcripts and dependencies identified here will serve as a foundational resource for the development of RNA-targeted precision medicines. Ultimately, this work highlights that the functional boundaries of the human genome extend beyond traditional gene definitions, opening avenues for exploring the complexity of eukaryotic biology and disease.

## MATERIALS AND METHODS

### Statistical analysis and design

Statistical analyses were performed using GraphPad Prism 10 software and R version 4.4.1. Differences between individual groups were analyzed for statistical significance using Welch’s *t* test, Wilcoxon rank-sum test, log-rank test, Fisher’s exact test, one-way ANOVA, or two-way ANOVA. *P < 0.05, **P < 0.01, and ***P < 0.001, and ****P < 0.0001. The data met assumptions of the statistical method used.

### Chimeric RNA prediction

The full chimeric RNA prediction pipeline developed for this study uses STAR-Fusion (v1.10.1) (*30*), Arriba (v2.2.1) (*32*), and FuSeq (1.1.4) (*33*) and requires the Biostrings R package (*62*) from Bioconductor. The pipeline was previously described in detail in a prior publication (*63*). Cell line RNA-seq data from the CCLE (*28*) (accession: PRJNA523380) was downloaded from the NCBI Sequencing Read Archive (https://ncbi.nlm.nih.gov/sra) using the SRA toolkit. RNA-seq data of patients with breast cancer from TCGA (dbgap accession phs000178) (*55*) were downloaded from NCI Genomic Data Commons using the GDC data transfer tool (*64*). For each sample, three chimeric RNA prediction programs were applied using GRCh38 v86 as the reference genome. STAR-Fusion was run using a max mate distance of 1000 to ensure detection of read-throughs, and a minimum junction support of 1, a minimum spanning frags of 0, and a minimum FFPM of 0.01 to increase sensitivity. All other settings were default. FuSeq was run with minJunctionDist set to 1000 and minGeneDist set to 0, with all other setting default. Arriba was run without the blacklist, read_through, and min_support filters, with everything else default. The outputs were standardized and concatenated using an R script, and then the gene annotations were standardized by converting the output ensemble IDs to the corresponding gene symbol from the GRCh38 version 86 gtf file. Additional filtering steps were applied to remove chimeras of the same gene, chimeras between paralogs, mitochondrial genes, immunoglobulins, and T cell receptors. Paralog genes were taken from Ensembl annotations and mitochondrial, immunoglobulins, and T cell receptor groups were taken from the HUGO Gene Nomenclature Committee annotations.

### Chimeric RNA quantitation

Chimeric and parental WT transcripts were quantified using a BLAST-based alignment strategy as described in previous literature (*35*). A comprehensive reference library was first constructed, comprising all predicted chimeric sequences and their corresponding parental WT transcripts. Chimeric templates were generated by concatenating every annotated 5′ parental exon sequence upstream of the donor site and every 3′ parental exon sequence downstream of the acceptor site, representing all possible pairwise isoform combinations. Raw sequencing reads (FASTQ) were converted to FASTA format and queried against this reference library using BLAST. To ensure high-confidence quantification, alignment results were filtered to retain only those reads that specifically spanned the splice junction windows of either the chimeric or parental sequences. Final counts were determined based on these filtered, junction-specific alignments. FFPM values were calculated by dividing the total chimeric RNA counts by the number of reads in the fastq file.

### Breakpoint classification

Breakpoint classification was carried out in R by analyzing the coordinates of the chimeric breakpoints. Chimeras were classified as “adjacent” if the breakpoint coordinates for both parental genes were located on the same strand of the same chromosome and within 70 kb of each other. Chimeras located on the same chromosome but on difference strands or outside 70 kb of each of were classified as “intrachromosomal.” Chimeras on separate chromosomes were classified as “interchromosomal.”

### Splicing predictions

Chimeric breakpoints were overlapped with exon annotations from the GRCh38v86 GTF in R. Breakpoints that fell within 2 base pairs of an exon boundary were classified as “E,” breakpoints that fell within an exon were labeled as “M,” and breakpoints that fell outside the exon boundaries (indicative of intergenic or intronic regions) were labeled as “O.” Chimeras with both breakpoints classified as “E” were labeled as “canonical,” those with a single exon labeled “E” were labeled “semicanonical,” and those with neither were labeled as “noncanonical.” Chimeras with O breakpoints were unquantifiable due to lack of transcript expression data and, therefore, removed from further analysis.

### Protein frame predictions

Protein-coding prediction were assessed by the predict_frame.py script from the FusionCatcher (*36*) prediction program, modified to accept out program output. Detailed predictions were then simplified into “in-frame,” “frameshift,” or “undefined.”

### SV predictions

DNA SV predictions generated from WGS were downloaded from DepMap (https://depmap.org/portal/data_page/?tab=allData&releasename=CCLE%202019&filename=CCLE_translocations_SvABA_20181221.xlsx). Chimeric RNAs were labeled as “fusion” if the full translocation with both parental genes was detected in the WGS data. They were labeled as “Single SV” if only a single parental gene had a detectable SV.

### Chimeric RNA functional screen

shRNA log fold changes (LFCs) and sequences preprocessed as described in McFarland *et al.* (*65*) were obtained from the DepMap repository under DEMETER2 v6 (https://depmap.org/portal/data_page/?tab=allData). The human reference genome (GRCh38) was indexed using Bowtie2 (v2.5.4) on UVA’s high-performance computing cluster. Probe sequences were aligned to the human genome using Bowtie2 with the parameters -k 10 -N 0 -L 21, and –no-1mm-upfront. Specifically, the alignment allowed up to 10 best matches per probe, disabled mismatches upfront, and used a seed length of 21 with zero mismatches permitted. Postalignment, probes aligning to multiple loci were removed to reduce off-target effects, and the resulting SAM files were converted to BAM then BED formats using samtools. Probe coordinates were intersected with Ensembl annotations (v86) to confirm correct gene mapping using bedtools. Final alignments were exported with genomic coordinates, strand orientation, and Ensembl gene identifiers.

Chimeric RNA-specific dependency scores were computed using an R pipeline integrating the chimeric RNA predictions with shRNA dependency scores from DepMap. Dependency datasets were combined into a unified matrix and then chimeric RNA predictions were parsed to extract 5′ and 3′ partner genes. Dependency scores for shRNAs targeting these genes were merged with fusion data, and shRNA probes were classified as upstream or downstream relative to the fusion breakpoint based on genomic coordinates and strand orientation. For each tractable fusion, a 5′ functional score and 3′ functional score were calculated by taking the difference between the mean dependency score across the chimeric breakpoint. For the 5′ parental gene, this was calculated as mean (UpstreamLFC) − mean(DownstreamLFC), and for the 3′ parental gene, this was mean(DownstreamLFC) − mean(UpstreamLFC). Each cell line was processed separately.

To estimate statistical significance, 10,000 random permutations of the shRNA LFC values were performed, and a normal distribution was fit to the resulting scores to model the null distribution of functional effects. *P* values were calculated using this fitted distribution to assess the probability of observing scores as extreme as those measured for each gene-chimera pair, followed by multiple testing correction with the Storey *q*-value method within confidence groups to estimate the FDR. Chimeras were labeled as dependencies if the functional score in either gene exceeded 0.5 and the *P* value was less than 0.05, and further classified as high confidence dependencies if the FDR < 0.5. All analyses were conducted using R version 4.3 with the tidyverse (*66*) and broom packages and executed on the UVA high-performance computing cluster (Rivanna) via SLURM.

### Overrepresentation analysis

Genomic and functional overrepresentation analysis was performed to characterize the features of identified chimeric transcripts. Transcripts were annotated according to four binary categories: Cancer Gene Census status (presence of a known oncogene or tumor suppressor in either parental position), identification as an established COSMIC fusion (*40*), predicted protein-coding potential, and the presence of underlying DNA SV evidence. To evaluate the enrichment of these features among growth-promoting hits, a two-tailed Fisher’s exact test was used, using the total pool of screened chimeric RNAs as the background set. The dataset was filtered to unique fusion–cell line pairs to ensure statistical independence and the analysis performed both as a combined analysis and stratified within confidence tiers. Results were quantified as OR with 95% confidence intervals (CI), with statistical significance defined as *P* < 0.05. All statistical modeling and data tidying were implemented in R.

### Long read sequencing analysis

Long read sequencing data in BAM format were downloaded from the ENCODE portal (https://encodeproject.org) by filtering for released experiments using long-read RNA-seq (assay_title=long read RNA-seq) (*51*) and then sorted using samtools. For each essential cis*-*SAGe chimera, reads were extracted from aligned BAM files using SAMtools by filtering for reads whose alignment spanned the chimeric breakpoint coordinates, determined by reads with a start position upstream of the 5′ breakpoint, an end position downstream of the 3′ breakpoint, and a spliced CIGAR string. Only primary alignments were retained. Unique read names passing this filter were used to retrieve all reads from the spanning fragment, which were written to per-fusion BAM files and indexed. Read counts were recorded per fusion per sample, and results were merged into a single count matrix. Results for select chimeras were visualized using Integrative Genome Viewer.

### Differential expression and GSEA

For each chimeric RNA, cell lines were stratified into high and low expression groups using the MAD. Raw gene counts were obtained from the DepMap portal (https://depmap.org/portal/data_page/?tab=allData&releasename=DepMap%20Public%2025Q3&filename=OmicsExpressionRawReadCountHumanAllGenes.csv). DEGs were identified using DESeq2 comparing the high expression and low expression groups. Gene set enrichment was performed using the fgsea function from the ClusterProfiler (*67*) R library against the Hallmark gene sets from MsigDB (*46*).

### siRNA knockdown and proliferation assay

Chimeric RNA-specific siRNAs were designed spanning the chimeric junction to selectively target the chimeric RNA in relation to parental genes. siRNAs were manufactured by Integrated DNA Technologies (IDT) using their dsiRNA chemistry. Cells were seeded into six-well plates with three biological replicates per condition at a density of 100,000 cells per well and allowed to incubate at 37°C with 5% CO_2_ for 24 hours. Cells were transfected using 9 μl of Lipofectamine RNAiMax (Invitrogen no. 13778150) and a final siRNA concentration of 15 nM. Cells were incubated at 37°C with 5% CO_2_ and harvested 72 hours later. Cell proliferation was measured by cell counting using a Cellecta cell counter with trypan blue correction.

### Chimeric RNA expression analysis

Chimeric RNA expression was measured by qRT-PCR. Cells were washed twice with phosphate-buffered saline (PBS) and RNA was extracted using an RNAeasy mini kit (Qiagen no. 74104) according to the manufacturer’s protocol. Following extraction, 200 ng of RNA was used for cDNA synthesis according to the manufacturer’s protocol (Abclonal no. RK20433). Expression was measured by qPCR using 10 μl of 2× SYBR Green Master Mix (Abclonal no. RK21203), 6 μl of water, 1 μl of forward primer, 1 μl of reverse primer, and 2 μl of cDNA. Reactions were performed with three biological replicates and two technical replicates. For chimeric RNA knockdowns, primers were designed targeting the chimeric junction, both parental junctions, and GAPDH loading control. For cell cycle regulators, primers were designed based on gene sequences from GRCh38v86. Data analysis was performed in Microsoft Excel using the ΔΔCT method normalizing to GAPDH, then to the siNC control. All primers were designed based on GRCh38 reference genome and manufactured by IDT.

### RNA-seq and differential expression analysis

*RRM2-C2orf48* was knocked down in HCT116 cells using junction-targeting siRNA as previously described. At 72 hours posttransfection, cells were harvested and total RNA was isolated using an RNeasy Mini Kit (Qiagen, catalog no. 74104). RNA samples were stored at −80°C before shipment to Novogene (Novogene Co. Ltd.) for library preparation and sequencing. mRNA was enriched from total RNA using poly-T oligo-attached magnetic beads. Following fragmentation, first-strand cDNA was synthesized using random hexamer primers, followed by second-strand cDNA synthesis. The resulting cDNA libraries were completed via end-repair, A-tailing, adapter ligation, size selection, and PCR amplification. Quantified libraries were pooled and sequenced on an Illumina NovaSeq X Plus platform using a paired-end 150-bp (PE150) sequencing strategy, yielding a minimum of 6 GB of raw data per sample.

Transcript abundance was quantified from the paired-end raw reads using Kallisto, and transcript-level counts were collapsed to gene level using tximport. Differential expression analysis was performed using DESeq2 in R to compare the knockdown and control groups. GSEA was conducted using the fgsea package against the MSigDB Hallmark gene sets and the KEGG database (*68*). Enriched KEGG pathways were subsequently visualized using the pathview R package.

### Western blot

Knockdown of chimeric RNAs was performed as previously described using three biological replicates. Cells were harvested 72 hours after knockdown for protein extraction. Cells were detached with 0.05% trypsin, pelleted at 300*g* for 5 min, and washed once with 1× PBS. Pellets were resuspended in RIPA lysis buffer (50 mM tris, 150 mM NaCl, 1% TritonX-100, 0.5% deoxycholic acid, 0.1% SDS, 1 mM EDTA, and 10 mM NaF) supplemented with protease and phosphatase inhibitors (1:100 dilution) and lysed by mechanical disruption, followed by incubation on ice for 30 min with intermittent vortexing. Lysates were pelleted by centrifugation at 14,000*g* for 30 min at 4°C. Protein concentrations were determined using the Pierce BCA Protein Assay Kit (Thermo Fisher Scientific no. 23225) and concentration was normalized to 20 μg.

Protein samples were diluted in Laemmli buffer for gel electrophoresis. Fresh 4× Laemmli buffer was supplemented with β-mercaptoethanol stock solution at a 1:10 ratio and mixed with lysates to 1× before denaturing at 95°C. Equal amounts of protein were loaded onto 12% 15-well bis-tris SDS-PAGE gels (Thermo Fisher Scientific no. NP0343BOX) with Precision Plus Protein Dual Color Standards (Bio-Rad no. 1610374EDU). Gels were assembled in the manufacturer’s running apparatus and run in a 1× Mops buffer at 120 V until adequate separation of bands was observed. Proteins were then transferred to nitrocellulose membranes (Whatman Protran no. 10401196) using a wet transfer system with NuPAGE Transfer Buffer. Transfers were performed at 100 V for 1 hour at 4°C.

Membranes were blocked in 5% nonfat dry milk in 1× TBST for 30 min. Membranes were cut to separate the protein of interest (p21, p53, and p27) from the loading control (B-actin) and probed separately. Primary antibodies were diluted 1:1000 in 5% milk in TBST, and membranes were incubated with the primary antibody solution overnight in the cold room on a rocker. Membranes were washed three times in TBST for 5 min each, then incubated with the appropriate horseradish peroxidase (HRP)–conjugated secondary antibody (rabbit or mouse) diluted 1:3000 in 5% milk in TBST for 1 hour at room temperature. Following additional washes in TBST, membranes were imaged with chemiluminescent detection using HRP substrate (Millipore no. WBKLS0500).

Band intensities were quantified using ImageJ. Briefly, the intensity of the band of interest and loading control (B-actin) and local background signal were measured and the background was subtracted from the band intensity. Normalized protein expression was calculated by dividing the background-corrected intensity of the target protein by the background-corrected intensity of the corresponding β-actin band. Intensity was then normalized to the siNC (nontargeting) control group. Statistical significance between the knockdown and control groups was calculated using a Welch’s *t* test.

### Cell cycle analysis by flow cytometry

Cell cycle distribution was analyzed by flow cytometry. Cells were transfected as previously described with three biological replicates and were harvested after 72 hours. Before harvesting, cells were treated with 10 μM 5-ethynyl-2′-deoxyuridine (EdU) (Sigma-Aldrich no. 9005854) for 30 min. Cells were then pelleted and washed with cold 1× PBS. All centrifugation steps were at 4°C at 500*g* for 10 min. Cells were fixed and permeabilized using the Foxp3/Transcription Factor Staining Buffer Set (eBiosciences no. 00-5523-00) according to the recommended intracellular staining protocol for nuclear antigens (Thermo Fisher Scientific BestProtocols: Staining Intracellular Antigens for Flow Cytometry). Briefly, cells were resuspended in 1× Foxp3 Fixation/Permeabilization working solution and incubated at 4°C in the dark for 30 min. Pellets were then washed twice in cold 1× Permeabilization Buffer. To detect EdU, a Click-It reaction was performed by incubating cells in EdU detection buffer [1 μM Alexa Fluor 647-azide (Molecular Probes no. A10277), 100 mM ascorbic acid, and 1 mM CuSO_4_ in 1× PBS] for 30 min at room temperature in the dark. Pellets were washed once with 1× FACS Staining Buffer (2% fetal bovine serum and 0.1% sodium azide in 1× PBS, pH 7.4) then resuspended in 1× FACS Staining Buffer with DAPI (Invitrogen no. D1306) and RNase A (Sigma-Aldrich no. R5125). Samples were run on the Attune CytPix (Life Technologies), and results were analyzed using FlowJo v10.10.0.

### Ribosome pulldown

To assess ribosome occupancy of the chimeric RNA, ribosomes were isolated by low-speed centrifugation using the Ribosome Extraction Kit (Bestbio, no. BB-36061). Briefly, 1 × 10^7^ to 2 × 10^7^ cells were washed with cold PBS, resuspended in 500 μl of cold Reagent A, and incubated on ice for 10 min before homogenization with a Dounce homogenizer. The lysate was cleared by sequential centrifugation (1000*g*, 11,000*g*, and 12,000*g* to 16,000*g* for 5 to 10 min each, 4°C) to remove cells, nuclei, and other organelles. Ribosomes were then aggregated by adding 125 μl of Reagent B to the supernatant and incubating overnight at 2° to 8°C, followed by pelleting at 10,000*g* for 20 to 45 min at 4°C. RNA was extracted using TRIzol (Invitrogen, no. 15596026) and ribosome-associated RNA was analyzed by RT-PCR and gel electrophoresis to determine ribosome occupancy of the chimeric RNA.

### Proteomics analysis

To validate the expression of alternative ORF translation products arising from fusion events, mass spectrometry (MS/MS) data from the CCLE cohort (*49*) were analyzed (https://massive.ucsd.edu/ProteoSAFe/dataset.jsp?task=02cd1b6a7c674f3ebdbed300b5d9aa57). Fusion transcript CDS sequences were reconstructed from chimeric RNA calls by mapping genomic breakpoint coordinates into CDS space using Ensembl GRCh38 release 86 annotations, selecting the primary isoform for each partner gene as annotated by MANE (*69*). Fusions where either breakpoint could not be placed on an annotated exon were excluded. The 5′ partner CDS upstream of the breakpoint was concatenated with the 3′ partner sequence from the breakpoint onward to assemble a full-length transcript. Assembled fusion sequences were translated in silico using Biostrings, then digested with the cleaver program using trypsin. Cleaved peptides were then filtered against the human proteome to remove non–fusion-specific peptides. A custom, localized proteomic search database was constructed by appending 51 target tryptic fusion peptide sequences derived from predicted intergenic splicing events to the canonical human reference proteome (*70*). Global peptide-spectrum matching was executed across 392 individual cohort runs using the high-throughput search engine MSFragger through FragPipe (v24) (*71*). Candidate target spectra were identified by matching experimental precursors to the theoretical tryptic sequences within a strict high-resolution instrument mass tolerance window of ±0.05 *m*/*z* (mass/charge ratio). For each localized spectrum query block matching a target fusion peptide, individual string matrices were programmatically scanned to extract core MS2 fragmentation metrics. These included the absolute spectrum of retention time, precursor charge state, and unique scan identification index. The pipeline systematically captured individual search engine scores, prioritizing the MSFragger hyperscore (representing peak correlation intensity) and the expectation value (*E* value).

### NMD analysis

Fusion transcript CDS sequences were reconstructed from chimeric RNA calls by mapping genomic breakpoint coordinates into CDS space using Ensembl GRCh38 release 86 annotations, selecting the primary isoform for each partner gene as annotated by MANE. Fusions where either breakpoint could not be placed on an annotated exon were excluded. The 5′ partner CDS upstream of the breakpoint was concatenated with the 3′ partner sequence from the breakpoint onward to assemble a full-length transcript. Junction reading frame was determined from CDS-space breakpoint positions. Assembled fusion sequences were translated in silico using Biostrings, and the first in-frame stop codon was designated the PTC. In-frame fusions were excluded from NMD evaluation as the ribosome is expected to terminate at or near the natural stop codon of the 3′ partner. For frameshift fusions, EEJ positions within the fusion CDS were derived from the 3′ partner exon structure, with the fusion junction itself included as an additional EEJ. A fusion was predicted as an NMD substrate if any EEJ downstream of the fusion junction was located more than 50 nucleotides 3′ of the PTC, consistent with the canonical NMD recognition rule. All analysis was performed in R.

### Subcellular localization analysis

Subcellular fractionation total RNA-seq data (cytosolic and nuclear fractions) for the K562 and HepG2 cell lines were obtained from the ENCODE Project (accessions ENCLB644EBX, ENCLB716GPJ, ENCLB581UDH, ENCLB941KGI, ENCLB014EDA, ENCLB758HRD, ENCLB278NDX, and ENCLB538THW) (*51*). Chimeric RNA fusions were predicted as previously described, and FFPM were calculated using FusionBlaster. For each chimeric RNA, FFPM values were averaged across replicates within each fraction, and a pseudocount of 0.001 was added to both fractions to allow calculation of fold change for fusions with zero counts in either compartment. The nuclear-to-cytosolic fold change was calculated as the ratio of average nuclear FFPM to average cytosolic FFPM, and log2 transformed to yield a log_2_FC for each fusion, with positive values indicating nuclear enrichment and negative values indicating cytosolic enrichment. Localization patterns for putative essential cis-SAGE chimeric RNAs were visualized as a heatmap generated with the pheatmap package in R.

### TCGA differential expression analysis

Expression changes in FFPM for chimeras and counts per million (CPM) for parental genes were calculated between primary tumor (*n* = 1059) and tumor-adjacent normal (*n* = 95) samples using the FusionBlaster pipeline, which quantifies both chimeric and parental expression based exclusively on junction-spanning reads, thereby avoiding ambiguity from shared sequence regions. Log_2_FCs were calculated for each essential cis*-*SAGe transcript between the two groups by log_2_((mean_FFPM_tumor + 0.00001)/(mean_FFPM_normal + 0.00001)). Statistical significance was evaluated using the Wilcoxon rank-sum test, with *P* values adjusted for multiple hypothesis testing via the Benjamini-Hochberg correction. Transcripts were classified as significantly differentially expressed if they exhibited an adjusted *P* value <0.05 and |log_2_FC| > 0.6.

### Survival analysis

Overall survival was modeled using the Kaplan-Meier method, with patients stratified into quartiles based on adjacent-class chimeric RNA burden. Clinical data for TCGA were compiled by Liu *et al.* (*72*). Survival disparities between the first (Q1) and third (Q3) quartiles were evaluated using a two-sided log-rank test. To identify individual chimeric transcripts with prognostic value, we used univariate Cox proportional hazards regression models using chimeric expression (FFPM) as a continuous covariate. To account for multiple hypothesis testing, *P* values were adjusted using the Benjamini-Hochberg correction procedure. All computational analyses were performed in R using the survival, survminer, and ggsurvival packages.

### Tau specificity score

To evaluate the distribution of chimeric RNA expression across breast cancer molecular environments, we calculated the Tau specificity score for each essential cis*-*SAGe transcript. Samples from the breast cancer cohort were first categorized by their PAM50 molecular subtype. For each chimeric RNA, we calculated the mean expression level across each subtype to generate a representative expression matrix. The degree of subtype-biased expression was then quantified using the Tau index (*73*, *74*). Tau scores range from 0 to 1, where 0 indicates constitutive expression across all subtypes and 1 indicates absolute specificity for a single subtype. Transcripts were classified as having high subtype specificity if they exceeded the established threshold of 0.8, as is standard. For each transcript, the “enriched subtype” was defined as the molecular group exhibiting the maximum mean expression. All statistical processing and visualization were performed in R version 4.3 using the tidyverse package.

### Cell proliferation assay

MDA-MB-436 cells were seeded at a density of 500,000 cells per well in a six-well tissue culture-treated plate. siRNA-mediated knockdown of chimeric RNAs was performed as previously described with two biological replicates per condition. Cells were harvested and counted 24 hours following transfection and cells were reseeded into a 96-well plate with three technical replicates per sample. Cells were incubated in a Sartorius Incucyte SX5 imager with bright-field images taken every 12 hours over a period of 5 days. Percent area covered was calculated by the Sartorius Incucyte analysis software and conditions were compared using a Welch’s *t* test at each time point.
