# Supplementary material for: Integrative discovery and functional dependency mapping uncover essential chimeric RNAs in the active transcriptome
Source: Sci Adv. 2026 Oct 30;12(44):eaei0901. doi: 10.1126/sciadv.aei0901 (PMC13721984; doi:10.1126/sciadv.aei0901)
Supplement: Supplementary file 1 [file sciadv.aei0901_sm.pdf]

Science Advances
AAAS

Supplementary Materials for

Integrative discovery and functional dependency mapping uncover essential chimeric RNAs in the active transcriptome

Samir Lalani *et al.*

Corresponding author: Hui Li, hl9r@virginia.edu

Sci. Adv. **12**, eaei0901 (2026)
DOI: 10.1126/sciadv.aei0901

This PDF file includes:

Figs. S1 to S11
Legends for tables S1 to S16

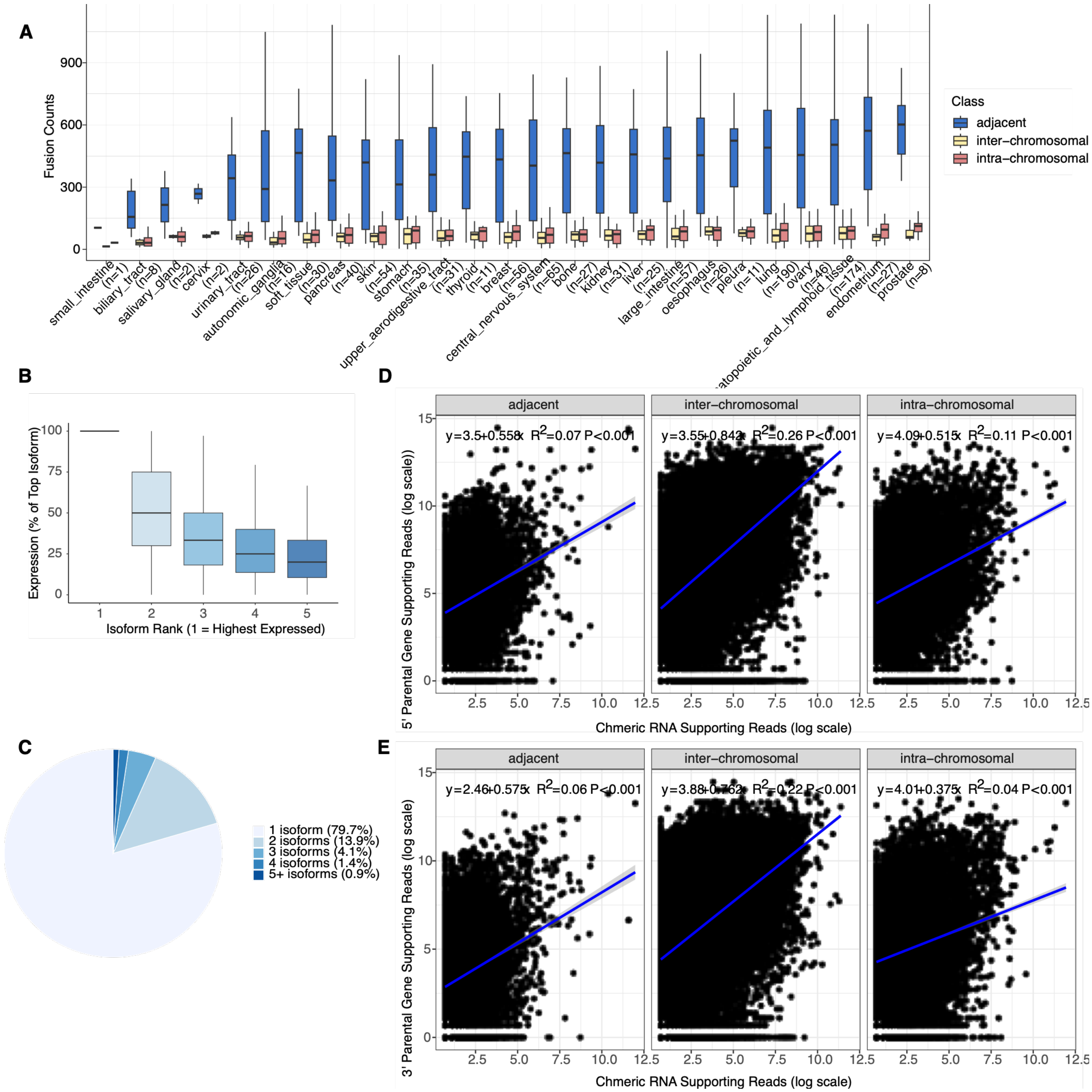
A
Fusion Counts
Class
adjacent
inter−chromosomal
intra−chromosomal
small_intestine (n=1)
biliary_tract (n=8)
salivary_gland (n=2)
cervix (n=2)
urinary_tract (n=26)
autonomic_ganglia (n=16)
soft_tissue (n=30)
pancreas (n=40)
skin (n=54)
stomach (n=35)
upper_aerodigestive_tract (n=31)
thyroid (n=11)
breast (n=56)
central_nervous_system (n=65)
bone (n=27)
kidney (n=31)
liver (n=25)
large_intestine (n=57)
oesophagus (n=26)
pleura (n=11)
lung (n=190)
ovary (n=46)
haematopoietic_and_lymphoid_tissue (n=174)
endometrium (n=27)
prostate (n=8)
B
Expression (% of Top Isoform)
Isoform Rank (1 = Highest Expressed)
C
1 isoform (79.7%)
2 isoforms (13.9%)
3 isoforms (4.1%)
4 isoforms (1.4%)
5+ isoforms (0.9%)
D
adjacent
inter−chromosomal
intra−chromosomal
y=3.5+0.558x R2=0.07 P<0.001
y=3.55+0.842x R2=0.26 P<0.001
y=4.09+0.515x R2=0.11 P<0.001
5' Parental Gene Supporting Reads (log scale))
Chmeric RNA Supporting Reads (log scale)
E
adjacent
inter−chromosomal
intra−chromosomal
y=2.46+0.575x R2=0.06 P<0.001
y=3.88+0.762x R2=0.22 P<0.001
y=4.01+0.375x R2=0.04 P<0.001
3' Parental Gene Supporting Reads (log scale)
Chmeric RNA Supporting Reads (log scale)

Supplemental Figure 1: Distribution, isoform diversity, and expression features of chimeric RNAs in cancer cell lines

(A) Number of detected chimeric RNAs per cell line grouped by tissue type and stratified by breakpoint class: adjacent (blue), intra-chromosomal (yellow), and inter-chromosomal (red). Significance measured by one-way ANOVA ($F = 1.68$, $P = 0.022$) comparing total chimeric RNA counts between each tissue type.

(B) Chimeric RNA isoform expression levels presented as a proportion of the primary isoform. The primary isoform is defined as the transcript with the highest expression in a cell line (rank 1), with each rank showing the next highest expressed isoform.

(C) Pie chart representing the number of different isoforms found for each chimeric RNA. Isoforms are defined as a chimeric RNA formed between the same 2 genes with multiple breakpoint pairings within a cell line. Transcripts with five or more isoforms were grouped together

(D-E) Scatter plots illustrating the association of chimeric RNA expression (x axis) with 5' (D) and 3' (E) parental gene expression, measured by FFPM. Panels are separated based on breakpoint class. Blue lines represent the linear regression fit with the line equation, R^2 value, and p-value indicated on each panel. For all comparisons $P < 0.001$.

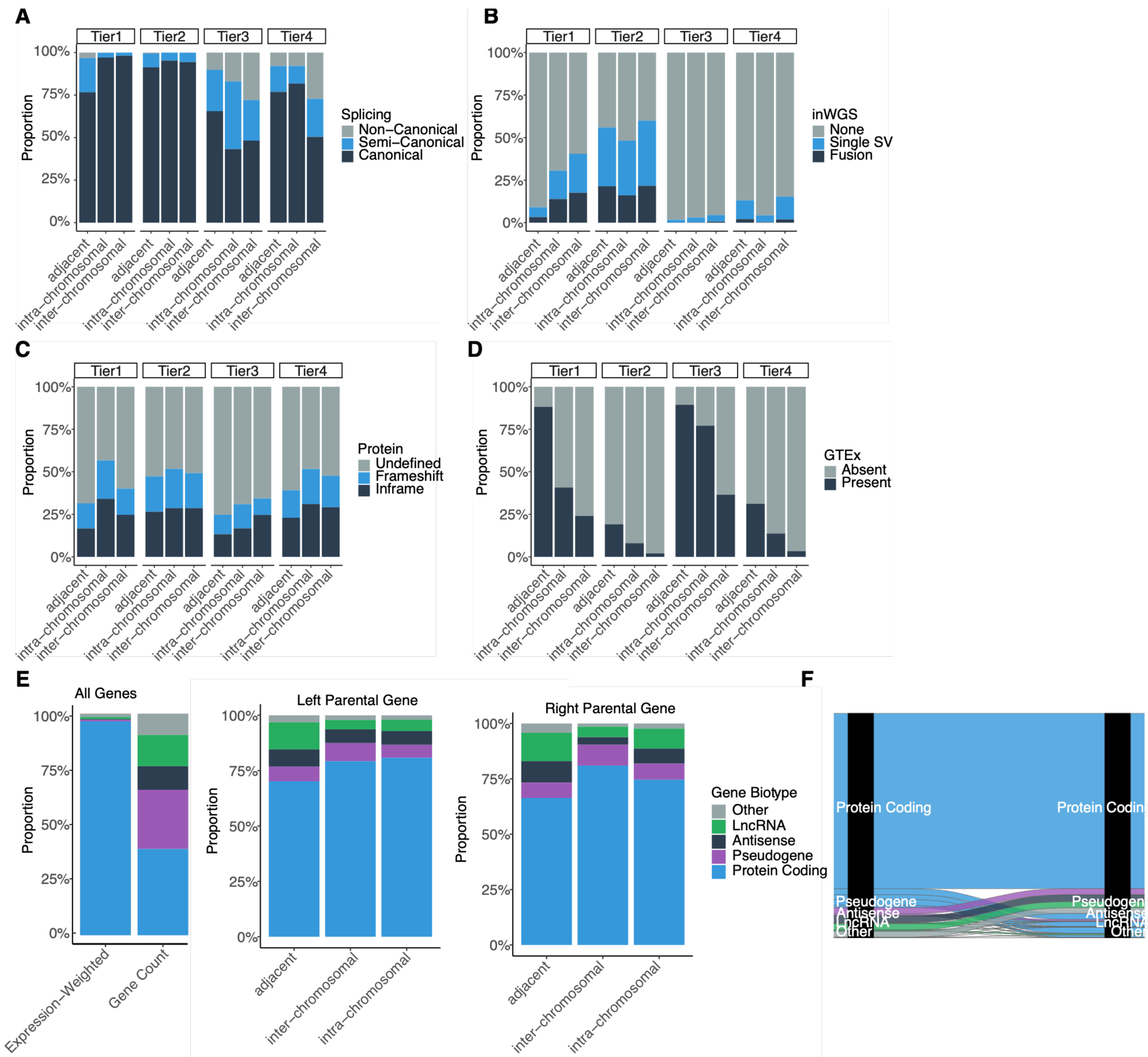

Supplemental Figure 2: Chimeric RNA traits and parental gene biotypes

(A) Splicing classification comparison of chimeric RNAs grouped by breakpoint and confidence class: canonical (both junctions on annotated exon boundaries), semi-canonical (one junction on an annotated exon boundary), and non-canonical (neither junction on annotated exon boundaries).

(B) DNA-level support for chimeric RNAs based on structural variant (SV) calls from whole-genome sequencing grouped by breakpoint and confidence class. Chimeras with rearrangements involving both parental genes were classified as "fusion," while those with a structural variant (SV) in only one parental gene were classified as "single SV."

(C) Proportion of chimeric RNAs predicted to encode in-frame or frameshifted proteins grouped by breakpoint and confidence class, based on whether the breakpoint preserves or disrupts the annotated reading frame. Chimeras with breakpoints outside known coding regions were classified as undefined.

(D) Proportion of chimeric RNAs previously detected in normal tissues from GTEx grouped by breakpoint and confidence class, defined as any fusion involving the same parental genes observed in at least one GTEx sample, regardless of recurrence or expression level.

(E) Comparison of gene biotype frequencies among all expressed genes in CCLE both expression weighted and overall (left), 5' parental genes (middle), and 3' parental genes (right).

(F) Alluvial plot illustrating pairing frequencies between 5' and 3' genes by biotypes.

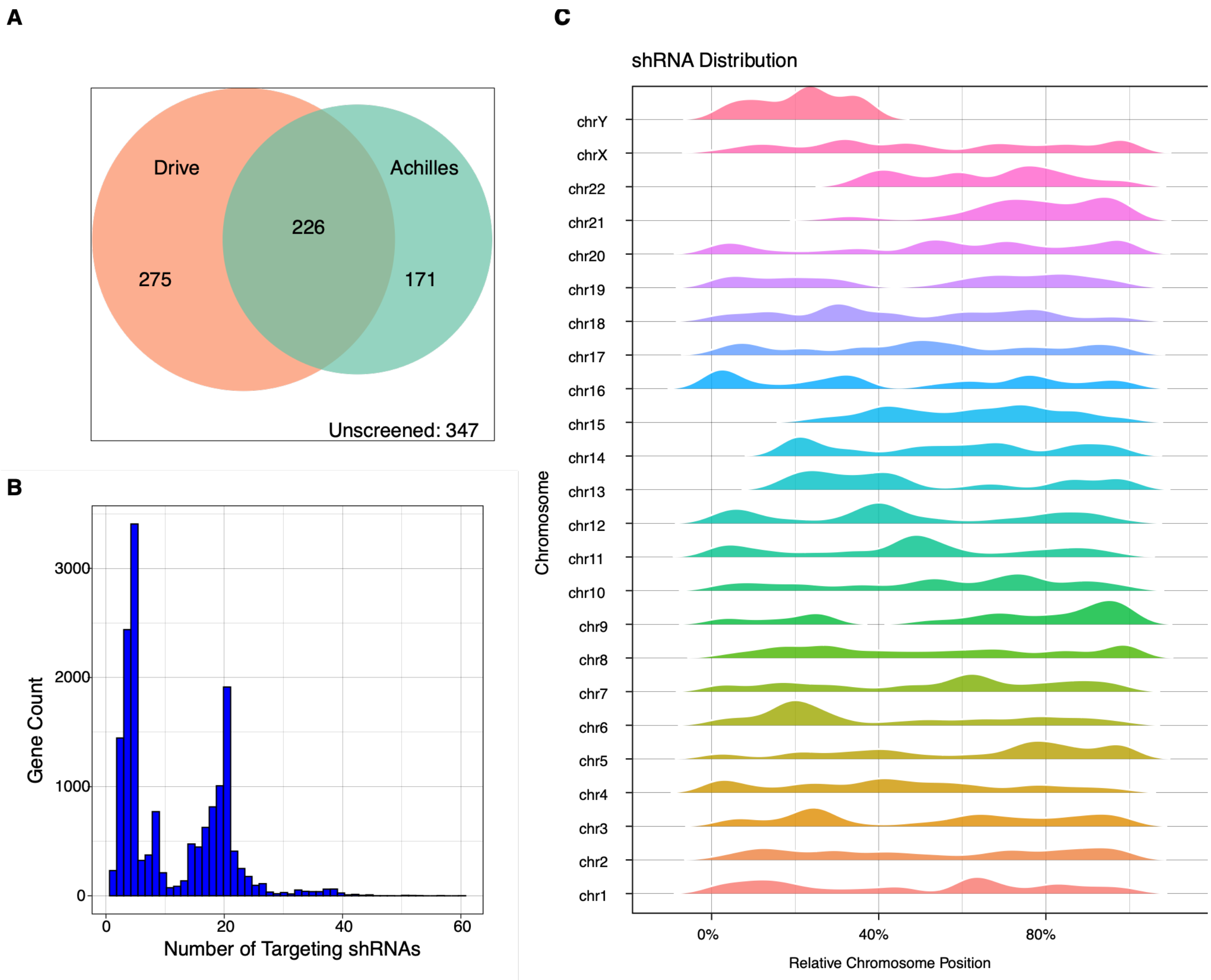

Supplemental Figure 3: Overview of RNAi screening dataset

(A) Venn diagram illustrating the intersection of cell lines investigated in the two RNAi dependency screening projects used in this study. Green represents Project Achilles (Tsherniak et al. 2017 (*22*)) and orange represents Project DRIVE (McDonald et al. 2017 (*23*)). The overlap region of 226 cell lines represents lines screened in both studies. 347 cell lines sequenced as a part of The Cancer Cell Line Encyclopedia were unscreened.

(B) Distribution of targeting probe counts per gene across the combined datasets. The observed bimodal distribution is attributable to distinct probe design strategies used by Project Achilles and Project Drive.

(C) Ridge plot displaying the density distribution of targeting probes along each chromosome. The x-axis represents genomic position relative to the total length of the respective chromosome.

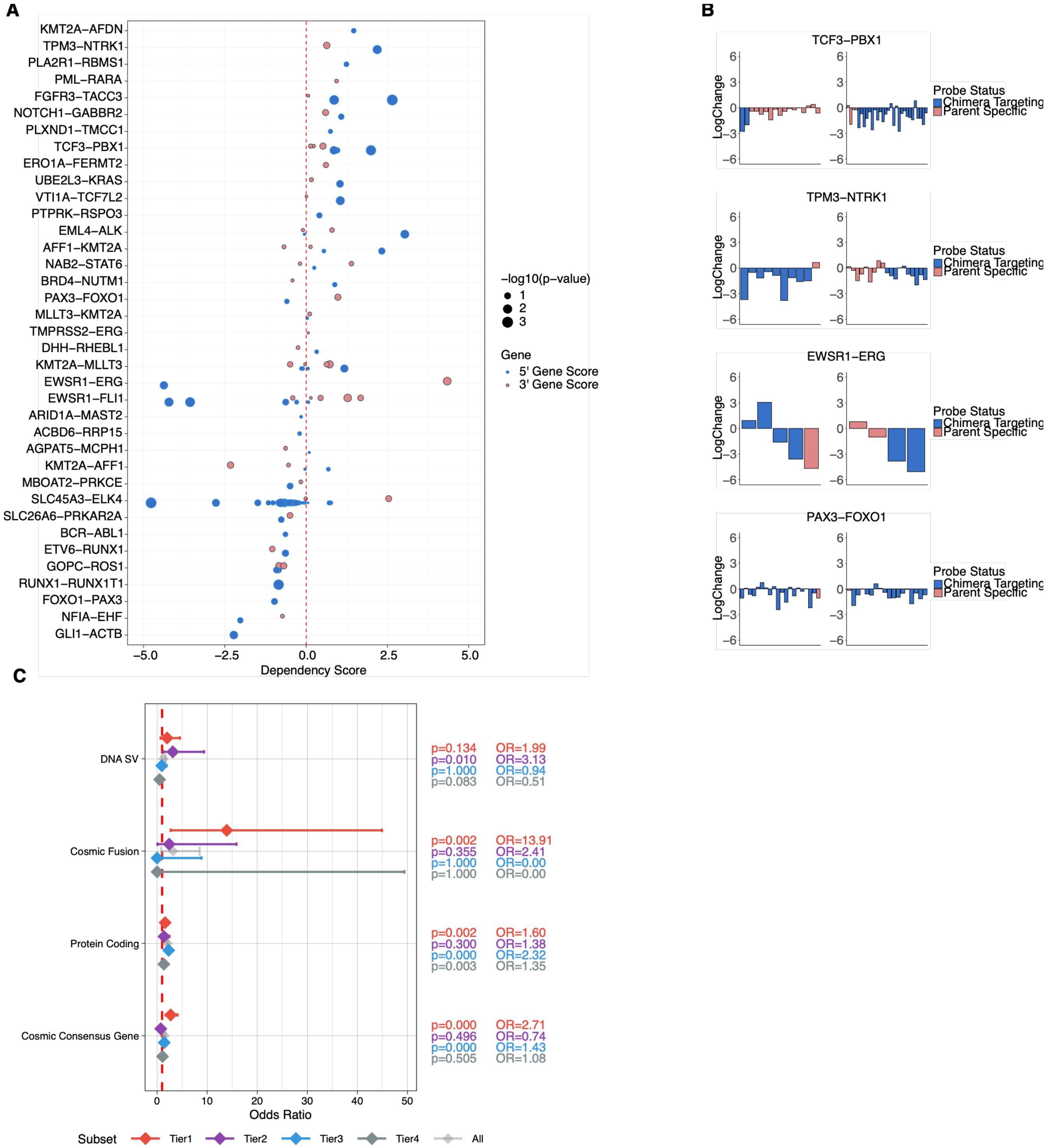
A
KMT2A–AFDN
TPM3–NTRK1
PLA2R1–RBMS1
PML–RARA
FGFR3–TACC3
NOTCH1–GABBR2
PLXND1–TMCC1
TCF3–PBX1
ERO1A–FERMT2
UBE2L3–KRAS
VTI1A–TCF7L2
PTPRK–RSPO3
EML4–ALK
AFF1–KMT2A
NAB2–STAT6
BRD4–NUTM1
PAX3–FOXO1
MLLT3–KMT2A
TMPRSS2–ERG
DHH–RHEBL1
KMT2A–MLLT3
EWSR1–ERG
EWSR1–FLI1
ARID1A–MAST2
ACBD6–RRP15
AGPAT5–MCPH1
KMT2A–AFF1
MBOAT2–PRKCE
SLC45A3–ELK4
SLC26A6–PRKAR2A
BCR–ABL1
ETV6–RUNX1
GOPC–ROS1
RUNX1–RUNX1T1
FOXO1–PAX3
NFIA–EHF
GLI1–ACTB
−5.0 −2.5 0.0 2.5 5.0
Dependency Score
−log10(p−value)
1
2
3
Gene
5' Gene Score
3' Gene Score
B
TCF3–PBX1
TPM3–NTRK1
EWSR1–ERG
PAX3–FOXO1
LogChange
6 3 0 −3 −6
Probe Status
Chimera Targeting
Parent Specific
C
DNA SV
Cosmic Fusion
Protein Coding
Cosmic Consensus Gene
0 10 20 30 40 50
Odds Ratio
p=0.134 OR=1.99
p=0.010 OR=3.13
p=1.000 OR=0.94
p=0.083 OR=0.51
p=0.002 OR=13.91
p=0.355 OR=2.41
p=1.000 OR=0.00
p=1.000 OR=0.00
p=0.002 OR=1.60
p=0.300 OR=1.38
p=0.000 OR=2.32
p=0.003 OR=1.35
p=0.000 OR=2.71
p=0.496 OR=0.74
p=0.000 OR=1.43
p=0.505 OR=1.08
Subset
Tier1
Tier2
Tier3
Tier4
All

Supplemental Figure 4: Analysis of known oncogenic gene fusions by RNAi dependency screen
(A) Dot plot depicting the functional scores for annotated oncogenic fusions in the Catalog of Somatic Mutations in Cancer (COSMIC) database detected and screened in the study. Blue points represent the functional score derived from the 5' gene, while red points represent the score from the 3' gene. Higher functional scores indicate a higher degree of dependency, reflecting a greater predicted effect on cell fitness following knockdown.
(B) Functional scoring profiles for additional representative COSMIC fusions. Bars represent scaled log2-fold changes (L2FC) across the transcript from 5' to 3' orientation, with fusion-targeting (blue) indicating shRNAs map to the chimeric transcript and parent-specific (red) indicating shRNAs mapping exclusively to parental genes. Negative L2FC represents a decrease in cell viability and positive represents an increase.
(C) Forest plot depicting enrichment of DNA structural variants, COSMIC fusion status, COSMIC parental gene status, and protein-coding potential among high confidence functional chimeras (functional score > 0.5, q value < 0.5) compared to all chimeras, separated by confidence tier. Diamonds indicate odds ratios and bars represent the 95% confidence intervals. Odds ratios calculated by two-tailed Fisher's exact test, *n* = 131,323 total chimeric RNA; chimeric RNA counts per category vary due to missing data.

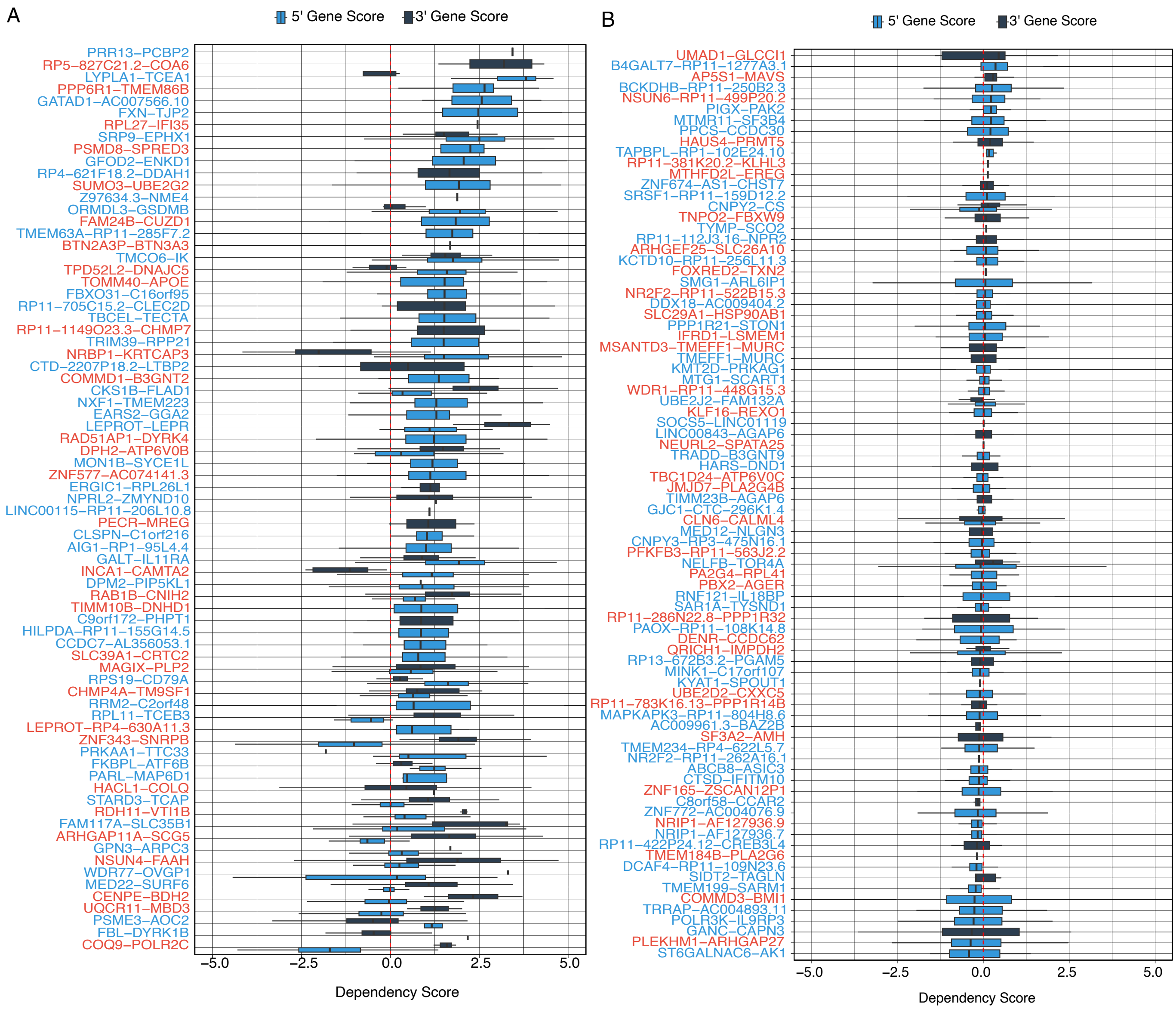

Supplemental Figure 5: Essential and non-essential cis-SAGe chimeric RNAs in CCLE

(A) Box plot showing calculated median functional scores for essential cis-SAGe chimeras across all screened cell lines. Blue bars represent functional scores for 5' parental genes, and black bars represent functional scores for 3' parental genes. Chimeric RNA names are colored by confidence tier, tier1 (red) or tier3 (blue)

(B) Box plot showing median functional scores for non-essential cis-SAGe chimeras across all screened cell lines. Blue bars represent functional scores for 5' parental genes, and black bars represent functional scores for 3' parental genes. Chimeric RNA names are colored by confidence tier, tier1 (red) or tier3 (blue)

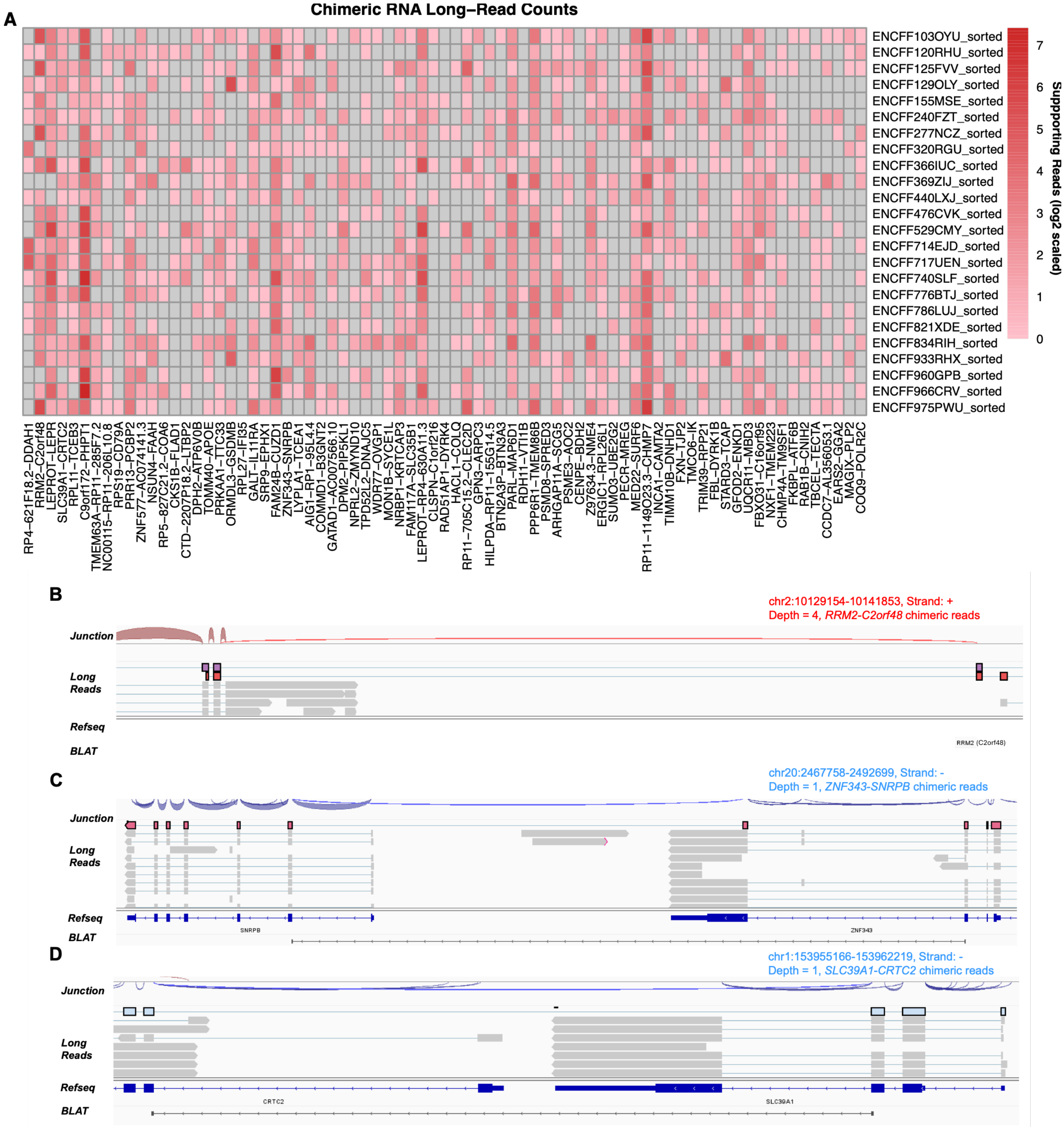

Supplemental Figure 6: Long read validation of cis-SAGe chimeric RNAs

(A) Heatmap showing long-read support for essential cis-SAGe chimeric RNAs across 16 ENCODE cell lines across 24 sequencing runs. Rows represent cell lines and columns represent chimeric RNAs. Color intensity indicates the log2-transformed number of supporting long reads; darker red indicates more supporting reads, and gray indicates no detected supporting reads.

(B–D) Genome browser views of representative long-read alignments for the cis-SAGe chimeric RNAs *RRM2–C2orf48* (B), *ZNF343–SNRPB* (C), and *SLC39A1–CRTC2* (D) in HCT116 cells. Tracks show junction coverage, long-read alignments, RefSeq gene annotations, and BLAT alignments of the junction sequence predicted by short read sequencing. Boxed regions highlight junction-spanning chimeric reads supporting each transcript.

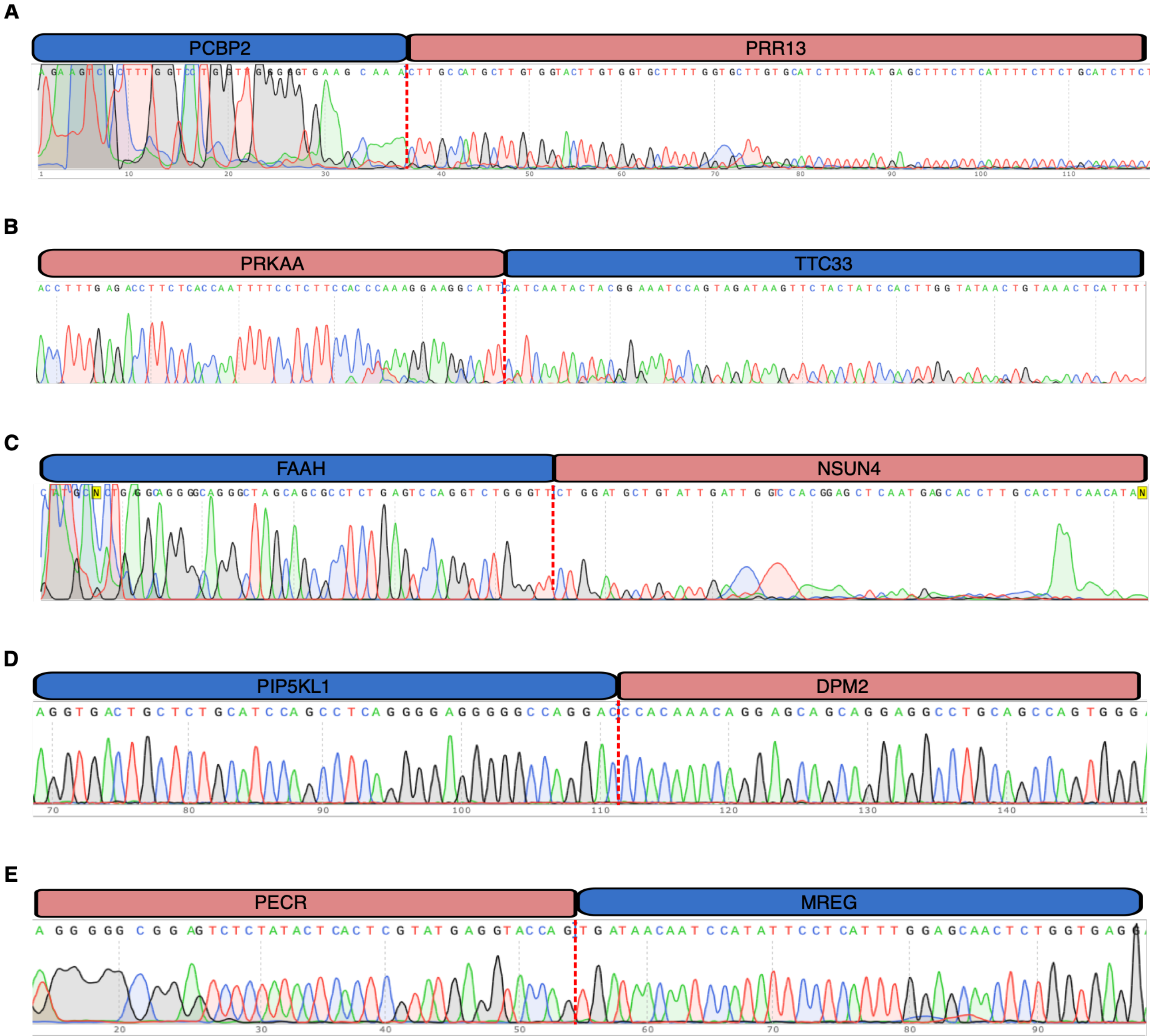

Supplemental Figure 7: Additional PCR validation of cis-SAGe chimeric RNAs

(A-E) Sanger sequencing traces of five additional chimeric RNAs amplified in HCT116 cells. These include *PRR13-PCBP2* (A), *NSUN4-FAAH* (B), *PRKAA1-TTC33* (C), *PIP5KL-DPM2* (D), and *MREG-PECR* (E). 5' genes are colored in red and 3' genes are colored in blue to show orientation of the transcripts. Directionality varies according to the primer used for Sanger sequencing.

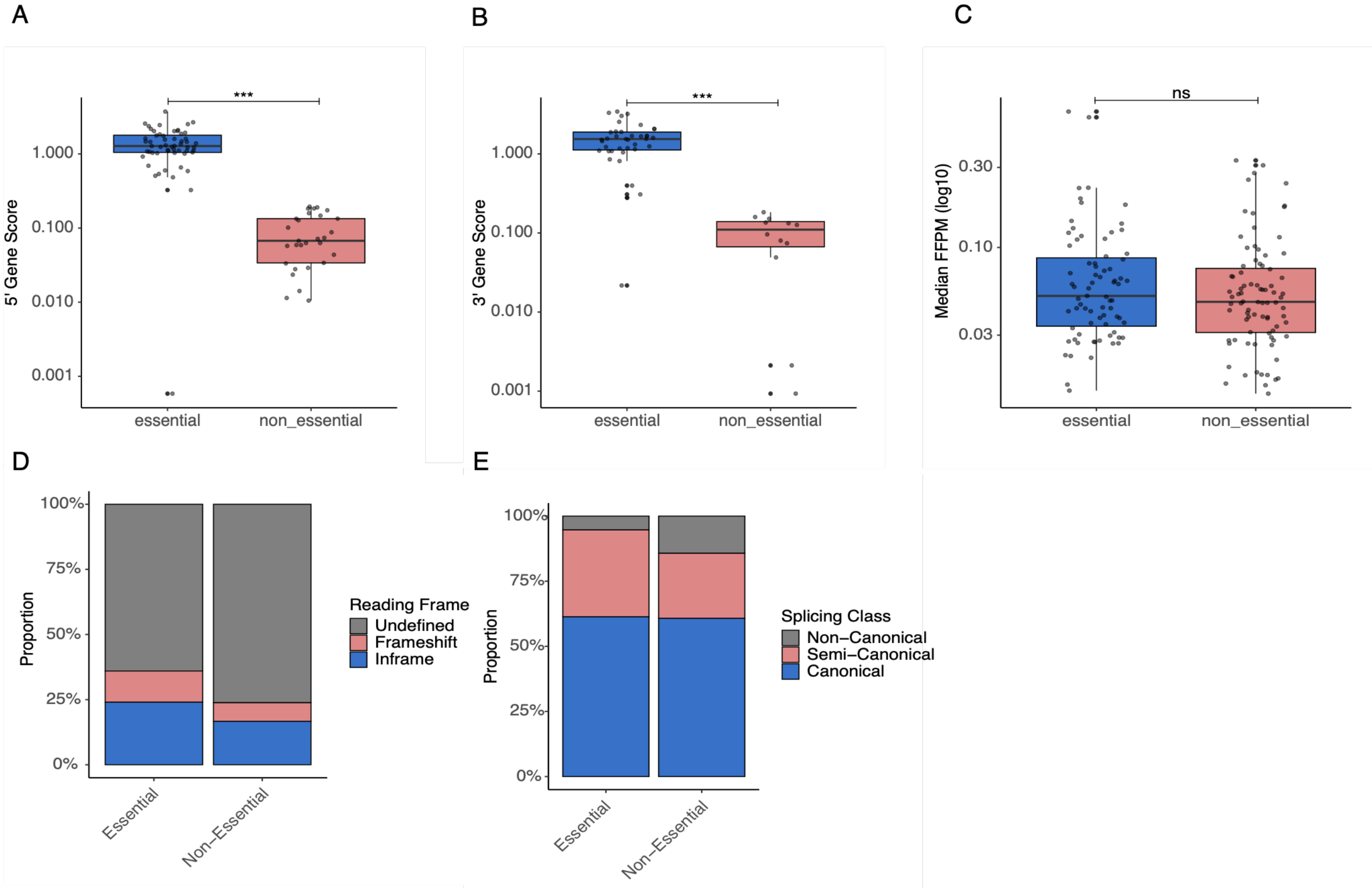

Supplemental Figure 8: Comparison of essential and non-essential cis-SAGe chimeric RNAs
(A–C) Box plots comparing essential (blue) and non-essential (red) cisSAGe chimeric RNAs by 5' gene score (A), 3' gene score (B), and median chimeric RNA expression in FFPM (log_{10} scale) (C). Statistical comparisons by two-tailed Welch's t-test (n = 75 essential, 86 non-essential, ****P* < 0.001).
(D–E) Stacked bar plots showing the proportion of essential and non-essential cisSAGe chimeric RNAs by reading frame class (in frame, frameshift or undefined) and splicing class (canonical, semi-canonical or non-canonical).

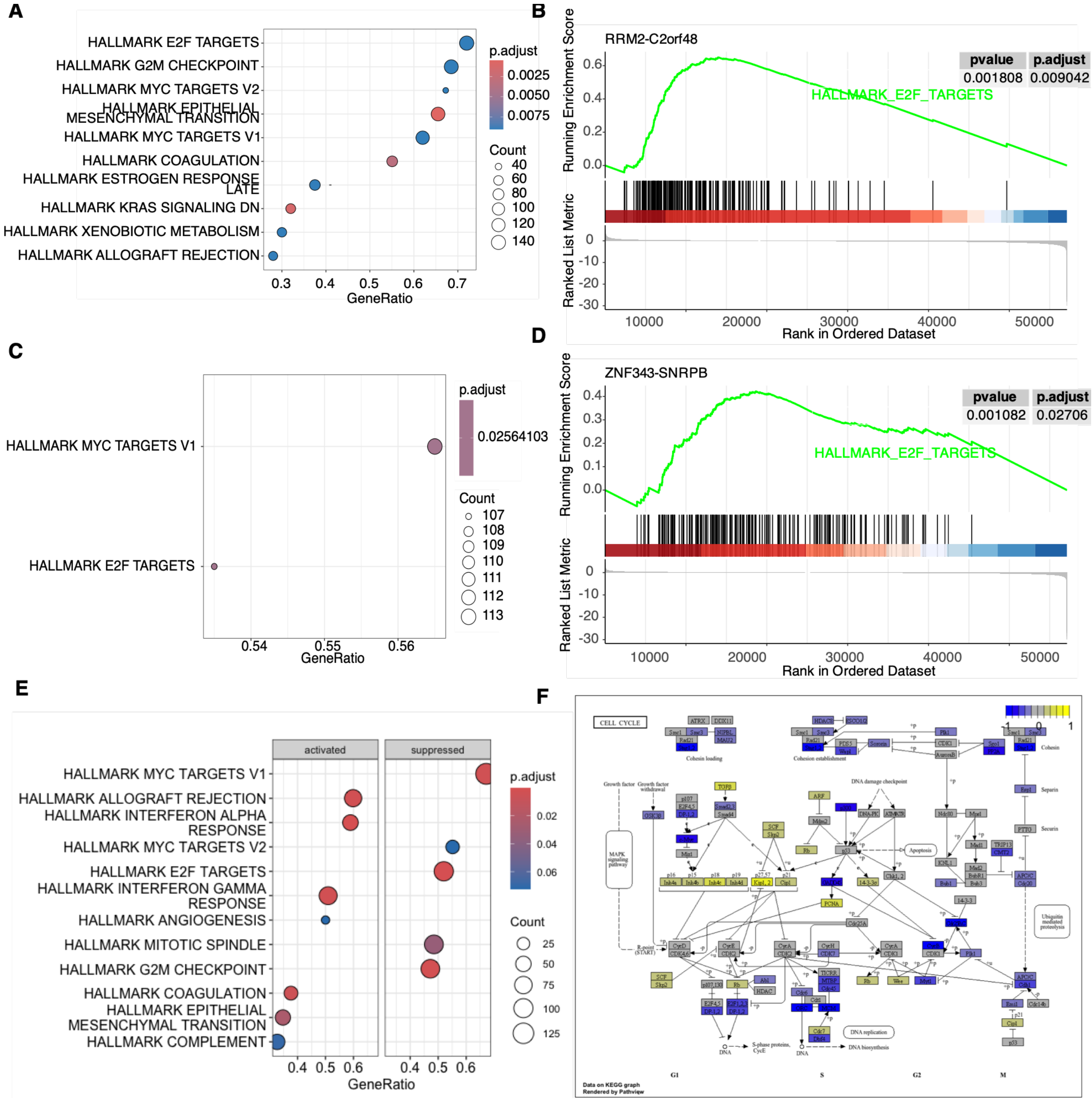

Supplemental Figure 9: Detailed GSEA results for RRM2-C2orf48 and ZNF343-SNRPB

(A-B) Significantly enriched Hallmarks pathways by gene set enrichment analysis in cell lines with high expression of *RRM2-C2orf48* (A) and *ZNF343-SNRPB* (B). Pathways shown have an adjusted p-value of < 0.05, dot size represents number of differentially expressed genes in each gene set.

(C-D) GSEA plot showing the enrichment of genes in the Hallmarks E2F targets genes set for cells with high expression of RRM2-C2orf48 (C) and *ZNF343-SNRPB* (D)

E) Significantly enriched Hallmarks pathways by gene set enrichment analysis following siRNA-mediated knockdown of *RRM2-C2orf48* and RNA-sequencing. Pathways shown have an adjusted p-value of < 0.05, dot size represents number of differentially expressed genes in each gene set.

F) KEGG pathway diagram showing differentially expressed genes in the cell cycle pathway following siRNA-mediated knockdown of *RRM2-C2orf48* and RNA-sequencing. Blue represents downregulation and yellow represents upregulation

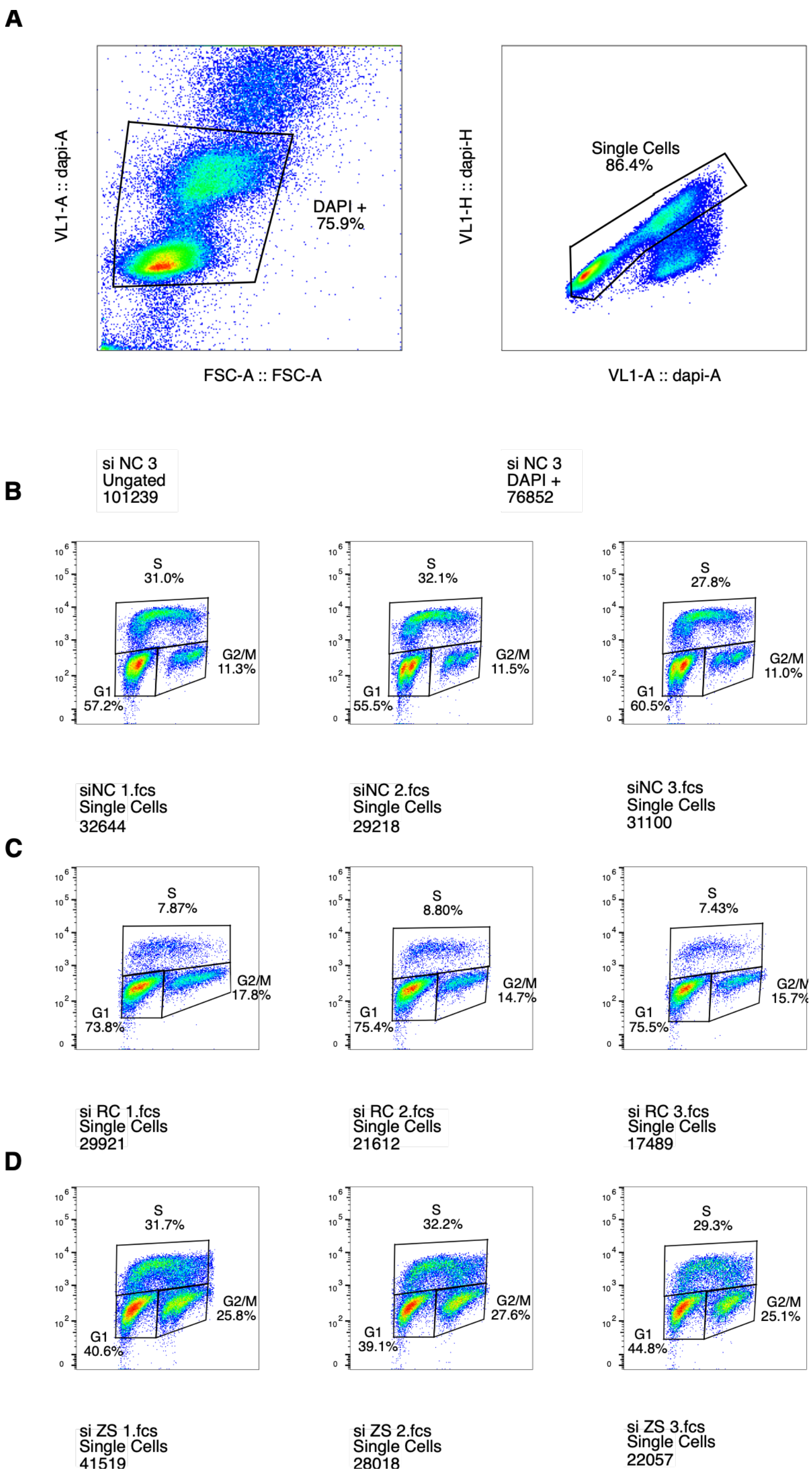

Supplemental Figure 10: Cell cycle flow cytometry extended data (From Figure 5)

(A) Gating strategy for flow cytometry to select for single cells: DAPI positive cells were gated to isolate live cells. From the DAPI positive population, single cells were then isolated

(B-D) Full flow cytometry analysis of cell cycle distribution using EdU incorporation and DAPI staining at 72 hours post-knockdown in HCT116 cells. Experiment was performed with 3 biological replicates per condition. Analysis compared the non-targeting control (B) to knockdown of *RRM2-C2orf48* (C) and *ZNF343-SNRPB* (D)

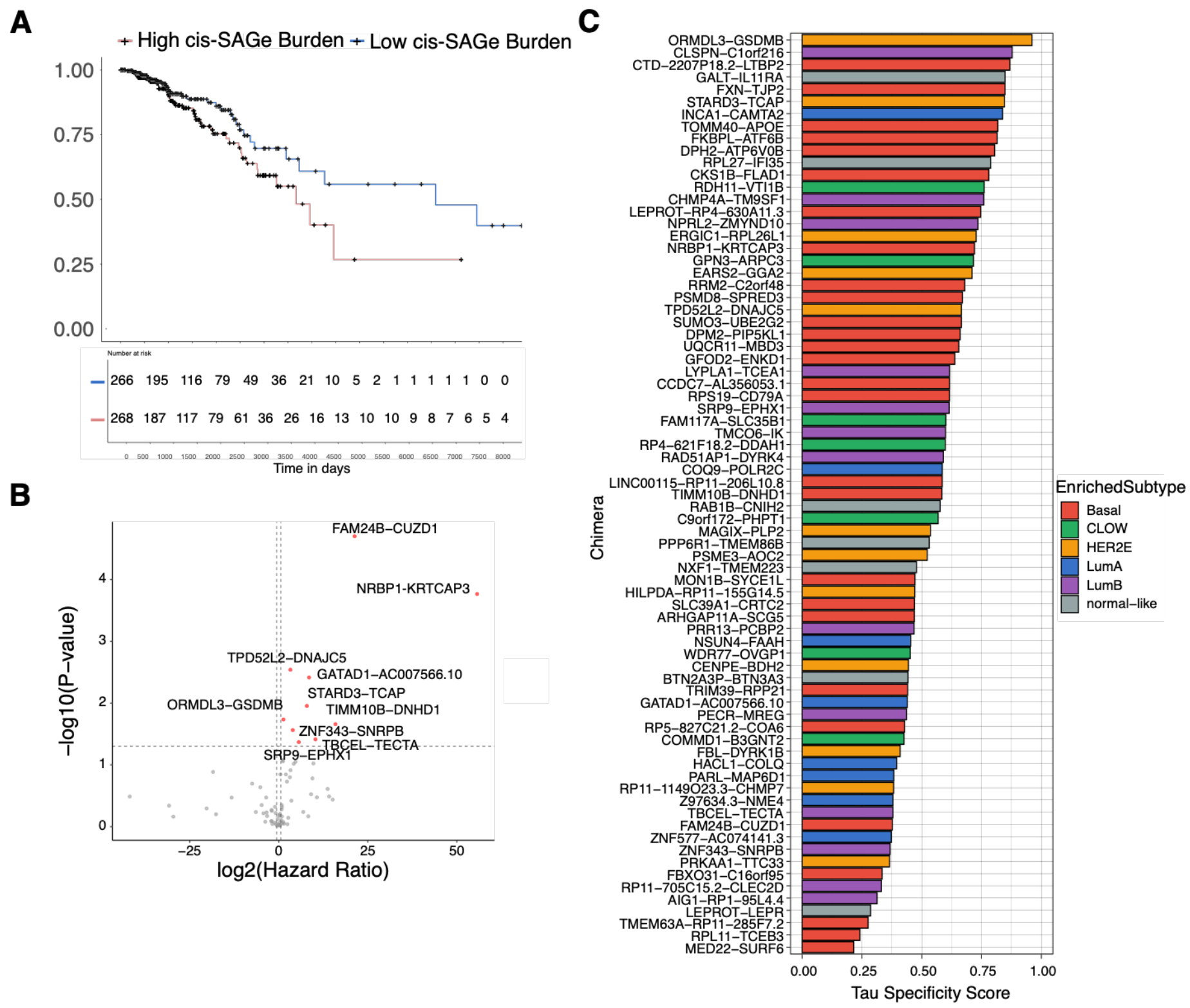

Supplemental Figure 11: Survival and subtype specificity of essential chimeric RNAs in breast cancer

(A) Kaplan-Meier overall survival curves comparing high cisSAGe-burden (Q3, red line) and low-burden (Q1, blue line). Statistical significance was determined using a log-rank test (n = 266 high, 268 low, *P* = 0.055).

(B) Univariate Cox proportional hazard model measuring the effects of chimeric RNA expression on overall survival, plotting the log2(Hazard Ratio) (x-axis) against the −log10(P-value) (y-axis). Chimeric RNAs (HR > 1, FDR =< 0.05) are highlighted in red.

(C) Tau specificity coefficient for each essential chimeric RNA, colored according to the most enriched clinical subtype. Tau > 0.8 denotes subtype-specificity.

Supplemental Tables- All tables are available through Zenodo at the follow link:
https://doi.org/10.5281/zenodo.23088886

SupplementalTable1	All chimeric RNA predictions from CCLE
SupplementalTable2	DepMap shRNA bowtie2 alignment
SupplementalTable3	Chimeric RNA functional screen results
SupplementalTable4	Essential cis-SAGe chimeric RNAs
SupplementalTable5	Non-essential cis-SAGe chimeric RNAs
SupplementalTable6	GSEA for essential chimeric RNAs
SupplementalTable7	GSEA for non-essential cis-SAGe chimeras
SupplementalTable8	Chimeric RNA support in ENCODE long-read data
SupplementalTable9	Mass spectrometry peptide evidence for chimeric RNAs
SupplementalTable10	Chimeric RNA nonsense-mediated decay (NMD) prediction
SupplementalTable11	Subcellular localization
SupplementalTable12	Chimeric RNAs in TCGA-BRCA
SupplementalTable13	BRCA clinical data
SupplementalTable14	Survival association of transcripts
SupplementalTable15	Subtype specificity of chimeras
SupplementalTable16	BRCA differential expression
